# A core mechanism for specifying root vascular patterning can replicate the anatomical variation seen in diverse plant species

**DOI:** 10.1242/dev.172411

**Published:** 2019-03-15

**Authors:** Nathan Mellor, John Vaughan-Hirsch, Britta M. C. Kümpers, Hanna Help-Rinta-Rahko, Shunsuke Miyashima, Ari Pekka Mähönen, Ana Campilho, John R. King, Anthony Bishopp

**Affiliations:** 1 Centre for Plant Integrative Biology/School of Biosciences, University of Nottingham, Sutton Bonington Campus, Loughborough LE12 5RD, UK; 2 Institute of Biotechnology, HiLIFE/Faculty of Biological and Environmental Sciences, University of Helsinki, Helsinki 00014, Finland; 3 Graduate School of Science and Technology, Nara Institute of Science and Technology, Nara 630-0192, Japan; 4 Research Center in Biodiversity and Genetic Resources, Department of Biology, Faculty of Sciences, University of Porto, 4485-661 Vairão, Portugal; 5 School of Mathematical Sciences/Centre for Plant Integrative Biology, University of Nottingham, University Park, Nottingham NG7 2RD, UK

**Keywords:** Multiscale modelling, Vascular pattern, Auxin, Cytokinin, Developmental biology, Root biology

## Abstract

Pattern formation is typically controlled through the interaction between molecular signals within a given tissue. During early embryonic development, roots of the model plant *Arabidopsis thaliana* have a radially symmetric pattern, but a heterogeneous input of the hormone auxin from the two cotyledons forces the vascular cylinder to develop a diarch pattern with two xylem poles. Molecular analyses and mathematical approaches have uncovered the regulatory circuit that propagates this initial auxin signal into a stable cellular pattern. The diarch pattern seen in *Arabidopsis* is relatively uncommon among flowering plants, with most species having between three and eight xylem poles. Here, we have used multiscale mathematical modelling to demonstrate that this regulatory module does not require a heterogeneous auxin input to specify the vascular pattern. Instead, the pattern can emerge dynamically, with its final form dependent upon spatial constraints and growth. The predictions of our simulations compare to experimental observations of xylem pole number across a range of species, as well as in transgenic systems in *Arabidopsis* in which we manipulate the size of the vascular cylinder. By considering the spatial constraints, our model is able to explain much of the diversity seen in different flowering plant species.

## Abstract (precis)

**Summary:** A gene regulatory network that generates a diarch vascular pattern in *Arabidopsis* roots can produce the range of patterns seen in flowering plants under different spatial constraints.

## INTRODUCTION

Patterning of organs involves the organization of cell fates within a tissue in space and time. Although the molecular networks that control pattern formation may be considered as deterministic, there is increasing evidence that other factors, including geometric constraints, environmental perturbations and stochastic effects, play key roles in the formation of stable patterns. In many processes, ranging from floral morphogenesis in plants (Álvarez-Buylla et al., 2008) to flagella length in Chlamydomonas (Kannegaard et al., 2014), stochasticity is required. Alterations in organ growth have also been shown to further the diversity in biological patterns, in systems based on morphogen gradients such as wing patterning in flies (Wartlick and González-Gaitán, 2011) and in polarity-based systems such as floral morphogenesis (Kennaway et al., 2011).

Roots represent an excellent choice of organ with which to study cellular patterning in plants because, when compared with other plant organs, the structures are both relatively simple and amenable to analysis via a range of microscopy techniques. Most cell division occurs within a zone known as the apical meristem that lies within the root tip; here, stem cells divide asymmetrically to generate initial cells for the different cell types. In vascular plants, the stele (or vascular cylinder) forms the central part of the root and is surrounded by outer layers, including the endodermis, cortex and epidermis. Cell identity is established already in the first daughter cells above the quiescent centre, and cell type-specific marker lines are expressed here, including markers such as TMO5 and AHP6, which define the xylem cell lineages (Schlereth et al., 2010; Mähönen et al., 2006a).

Xylem forms in distinct poles at the periphery of the stele, with phloem and parenchymatic cells located in between. The xylem cells undergo thickening of the secondary cell walls, and eventually senesce to become conduits that allow the transport of water and other components between organs. Many plant species also undergo an additional process of secondary growth, whereby cell divisions in the cambium cause the organ to thicken and vascular pattern to be re-specified. The number of xylem poles established during the primary growth phase varies, both between species, between individuals within a species and between roots of an individual plant. Previous researchers have observed a close correlation between stele size (diameter of the stele) and pole number during primary growth in a variety of plant species, including, lycopods, ferns and angiosperms (Wardlaw, 1928). This work laid the groundwork for what became known as the ‘size factor’ theory, in which the structural relationship between morphology and size was proposed to be a significant factor in determining the structure of roots.

**Fig. 1.**
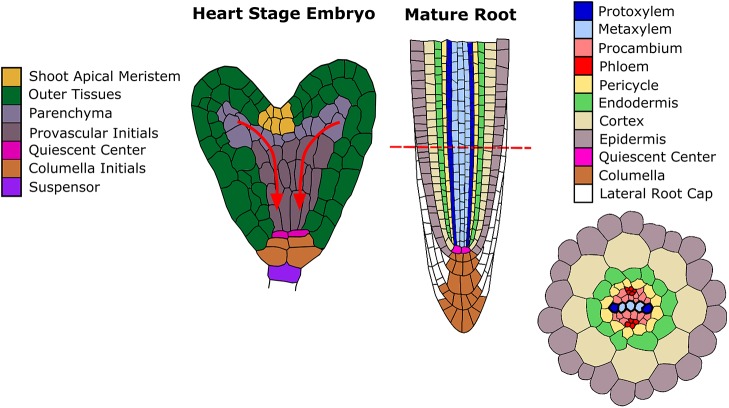
**Cell fate specification in *Arabidopsis thaliana*.** A schematic representation of the root apical meristem of the growing root, orientated so that the two xylem poles are visible. Alongside is a heart-stage embryo with the provascular cells indicated. The arrows show an approximation of the path that auxin travels into the root pole.

**Fig. 2.**
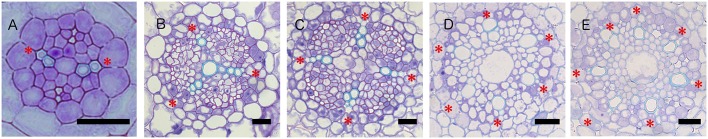
**The diversity of vascular pattern in different plant species.** (A) The model plant *Arabidopsis thaliana* contains two xylem poles. (B) *Lotus japonica* typically has three xylem poles. (C) *Medicago truncatula* typically has four xylem poles. (D,E) Monocotyledonous plants typically have a greater number, shown here are rice (kitaake) primary roots with six (D) and seven (E) poles. Samples have been stained with Toluidine Blue to highlight cell walls. Xylem poles are indicated with adjacent asterisks for clarity. Scale bars: 20 μm. The cross-sections were taken ∼4 mm from the tip for *Arabidopsis*, ∼2 cm from the tip for both *Medicago* and *Lotus*, and ∼3 cm from the root tip for rice.

More recently, the root vascular cylinder of the model plant *Arabidopsis* has been exploited as a model for pattern formation. Through the use of genetically encoded marker genes and advances in microscopy, the specification of the vascular pattern has been traced back through embryogenesis (Fig. 1). Specification of xylem cell fate constitutes a symmetry-breaking event that transforms a near radially symmetric root into a bisymmetric structure, with two xylem poles arranged in a diarch pattern (Figs 1 and 2A).

The diarch vascular pattern is driven through an early asymmetry in the signalling domains of two phytohormones, auxin and cytokinin (Mähönen et al., 2006a; Bishopp et al., 2011), in which cells with high auxin output differentiate as xylem. Several key interactions between these hormones specify distinct domains of hormonal signalling. The auxin response promotes the transcription of the cytokinin signalling inhibitor *ARABIDOPSIS HISTIDINE PHOSPHOTRANSFER PROTEIN 6* (*AHP6*) (Mähönen et al., 2006a; Bishopp et al., 2011). Cytokinin signalling modulates the activity of a group of auxin transport proteins known as PINFORMED proteins (PINs) (Gälweiler et al., 1998; Ruzicka et al., 2009; Bishopp et al., 2011; Pernisova et al., 2016). An additional feedback loop exists in which auxin signalling promotes cytokinin biosynthesis via activation of the *LONELY GUY4* (*LOG4*) enzyme (De Rybel et al., 2014). Although the domain of high auxin response extends throughout the xylem axis, the formation of protoxylem is restricted to the marginal positions. This is due to a group of class III HD-ZIP transcription factors, including the gene *PHABULOSA* (*PHB*), which acts in a dose-dependent manner to prevent protoxylem formation and *AHP6* expression in the central part of the root (Carlsbecker et al., 2010). This cytokinin-based patterning mechanism of specifying vascular pattern seems specific to the root, as shoot models of periodic auxin distribution can pattern vascular bundles solely via activity of auxin influx and efflux proteins (Fàbregas et al., 2015).

A number of theoretical studies have investigated this mechanism for generating stable vascular patterning in *Arabidopsis* roots (De Rybel et al., 2014; Muraro et al., 2014; el-Showk et al., 2015), with a consensus between the three models recently being drawn (Mellor et al., 2017). Collectively, these models support the hypothesis that the nonlinear feedback between auxin and cytokinin can transform an initial heterogeneity into the stable diarch pattern seen in *Arabidopsis*. Although different inputs have been modelled, experimental evidence points to an input of auxin coming from the two cotyledons during embryogenesis, as auxin signalling output is highest in the vascular initials that subtend the cotyledons (De Rybel et al., 2014). As the mature plant develops, cells within this embryonic root pole go on to form the hypocotyl. Consistently, mutants with altered cotyledon number have irregular numbers of xylem poles within the hypocotyl (Help et al., 2011).

Although these studies provide a feasible mechanism for generating the root vascular pattern seen in *Arabidopsis*, they cannot explain the variation in the patterns seen in many other species. Most dicot roots typically display three or four xylem poles during primary development (Fig. 2B,C), while monocots typically display five or more xylem poles (Fig. 2D,E) (Esau, 1965). These deviations suggest a clear disjunction between the final number of vascular poles and the number of cotyledons, and although the evidence is strong for the cotyledons having a role in establishing the initial vascular pattern seen in embryonic *Arabidopsis* roots, an alternative mechanism must be in place to pattern roots with three or more vascular poles. Previous research sheds light onto what this mechanism may be.

In the 1950s George Torrey used surgical techniques to manipulate *Pisum sativum* (pea) roots and follow the changes in vascular patterning. In such studies, 0.5 mm root tips were excised and allowed to regenerate in a synthetic medium, reaching a length of up to 60 mm within 1 week (Torrey, 1954). The apical pattern of isolated pea roots grown in culture was largely unaffected, but the diameter of these roots tended to decrease (Torrey, 1955). In control samples, *Pisum sativum* consistently showed a triarch vascular pattern; however, in the cultures prepared from excised root tips, a number of plants showed a reduction in the number of poles, with either diarch or monarch vascular patterns present. By correlating the root size with the number of vascular poles, Torrey showed a correlation between pole number and the size of the vascular cylinder at the time of pattern initiation (i.e. the apical meristem), but not with the final size of mature roots. As these patterns were specified in excised roots lacking input from either the older mature tissues or from the apical region of the plant, Torrey concluded that the root apical meristem of pea roots was self-determining and capable of self-patterning. In more modern terminology, we suggest that this points towards *de novo* patterning as an emerging property of the regulatory networks operating within the meristem.

Recent studies into the regulation of root vascular pattern have focused on pattern formation of the primary root in *Arabidopsis* and, owing to the limited and consistent size of these roots, have therefore not fully explored the role that spatial constraints can play in determining vascular pattern. In this article, we propose a vascular patterning mechanism consistent with both the modern molecular studies and the classic anatomical and surgical studies. Our results are consistent with recently published work suggesting that, in *Arabidopsis*, the cotyledons are used as a mechanism to determine the initial embryonic pattern (de Rybel et al., 2014; Help et al., 2011); however, we propose that vascular patterning can be set or re-established as an emergent property of the interaction between auxin and cytokinin. In this context, spatial constraints and stochastic elements play a significant role in directing the final pattern.

## RESULTS

### Root vascular patterning is a dynamic process that can be re-specified during organ growth

**Fig. 3.**
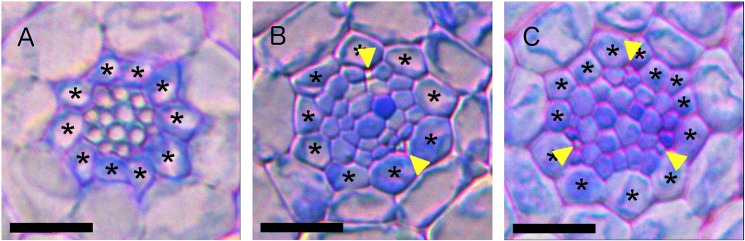
**Vascular patterning can be set *de novo* in *Arabidopsis* roots independently of heterogeneous auxin input.** (A) *Arabidopsis wol* mutants have roots with a reduced number of vascular cells, all of which have high auxin response and differentiate as xylem. (B,C) Post-embryonic rescue of the *wol* phenotype using the *pCRE1::XVE*≫*CRE1* line results in the establishment of a diarch or triarch vascular pattern after 3 days induction. Samples have been stained with Toluidine Blue to highlight cell walls. At this stage, the xylem is not completely lignified and is hard to visualize, therefore we mark phloem poles with yellow arrowheads to indicate the pattern. Pericycle cells are indicated with asterisks for clarity. Scale bars: 20 μm. See also Help-Rinta-Rahko (2016).

Recent molecular and theoretical studies of vascular patterning have focused on *Arabidopsis* primary roots and require further thought to interpret them in context with the earlier work by anatomists such as Wardlaw and physiologists such as Torrey. The model is that of *Arabidopsis*: the number of xylem poles is specified through a heterogeneous input of auxin from the cotyledons. As the post-embryonic root grows, the two xylem poles are maintained. However, in order to explore the process of vascular specification in *Arabidopsis* during post-embryonic root development, we first considered the maintenance of the embryonic pattern. In particular, it was unclear why the diarch pattern that was established during embryogenesis persisted in the growing root. One hypothesis was that once an embryonic pattern has been determined, *Arabidopsis* roots are unable to specify alternative vascular patterns. Another hypothesis is that the patterning mechanism actively drives the specification of a diarch pattern due to the relatively small size of the root. We developed an inducible transgenic system to test whether vascular pattern could be reset post-embryogenesis in *Arabidopsis*. To achieve this, we exploited the (*woodenleg*) *wol* mutant. This has a mutation in the CHASE domain of the *CYTOKININ RESPONSE1* gene (*CRE1*) that confers a constitutively active phosphatase activity that mimics the receptor in the cytokinin-unbound form (Mähönen et al., 2000, 2006b). Consequently, *wol* mutants have severely impaired cytokinin response in the root, and have a radially symmetric pattern of auxin response in which all vascular cells differentiate as protoxylem (Mähönen et al., 2006b; Bishopp et al., 2011). We created transgenic lines that could complement the *wol* mutant, by driving a functional copy of CRE1 in an inducible manner under its own promoter pCRE1::XVE≫CRE1 in the *wol* background. Non-induced plants resembled the *wol* mutant (Fig. 3A), but when we induced the transgene by germinating plants with estradiol, after 3 days we saw the re-generation of undifferentiated cells at the root tip and saw the *de novo* specification of vascular pattern in these tissues (Fig. 3B,C). While the wild-type *Arabidopsis* root invariably has a diarch vascular pattern, in the complementation line we saw instances of both diarch and triarch patterns being established. This demonstrates that vascular patterning can be set *de novo*, independently of the embryonic input provided by the cotyledons.

These results suggest that a molecular network exists within *Arabidopsis* that can set vascular patterning autonomously. Such a system would provide a mechanism whereby even if the initial vascular pattern was established during embryogenesis, it could be re-set in growing roots depending on organ size or on environmental conditions. Such a system would de-couple root vascular patterning from cotyledon number and may explain the wide variety of vascular patterns specified in the roots of other plant species.

### The interplay between genetic regulation and organ size determines vascular patterning in a static model

**Fig. 4.**
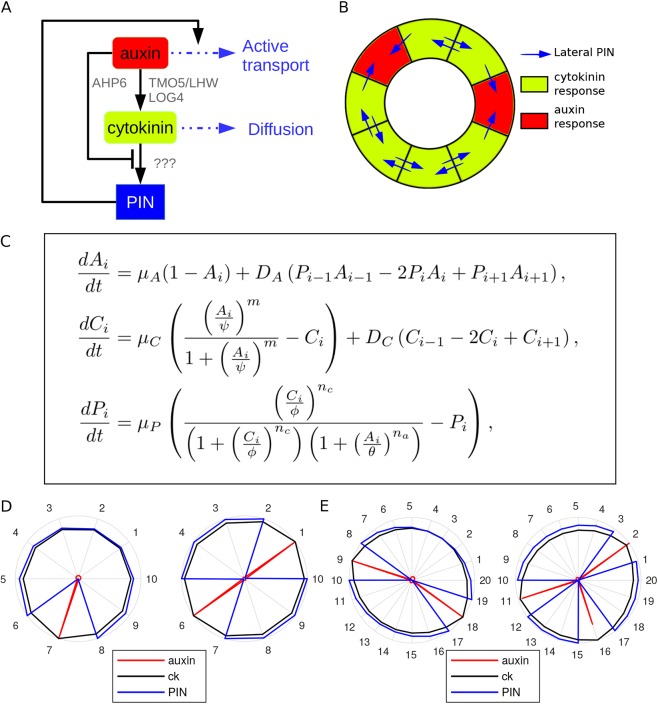
**A predictive model of vascular patterning.** (A) Schematic diagram showing network configuration. The black lines represent interaction between individual components; the blue lines represent transport of components between cells. (B) An example of the model template shown in its spatial context, with PIN activity (blue arrows) being dependent on hormonal output. (C) Model equations. *A_i_* represents auxin in cell i, *C_i_* represents cytokinin in cell i, *P_i_* represents PIN in cell i. See supplementary Materials and methods for details and formulation. (D,E) Representative steady-state predictions from the static model (for parameter values, see Table S2). The level of each component is shown on a relative scale between 0 and 1. Auxin is shown in red, cytokinin in black and PIN in blue. A high auxin response is indicative of sites where xylem will form. (D) Ten initial cells results in one or two poles. (E) Twenty initial cells results in two or three poles.

To test whether the molecular network that has been described for *Arabidopsis* can specify pattern as an emergent property and to investigate factors controlling final form, we developed a holistic model that can simulate pattering in a range of species. We consider three essential components only (auxin, cytokinin and PIN), rather than explicitly modelling individual gene products (Fig. 4A,C). Auxin represents both the molecule itself and the mechanism through which it is perceived. Rather than explicitly modelling an AHP6-like component that represses cytokinin signalling, we simplify the network so that auxin represses cytokinin signalling directly. Likewise, we make the simplification that auxin promotes the production of cytokinin directly, without including any intermediate components (such as the LOG genes). Cytokinin represents only the molecule itself, as auxin has the dual antagonistic effect of promoting the synthesis of cytokinin while repressing the cytokinin response, which we model through the interaction of auxin with PIN. We consider a generic PIN to be induced by cytokinin, with feedback through auxin. Finally, as an auxin efflux transporter, the presence of PIN in a cell enhances the unidirectional transport of auxin out of that cell. Like all previous models, we do not explicitly model the process of vascular differentiation, but interpret spikes of high auxin response as indicators of cells primed to differentiate as xylem.

To approximate the vascular cells immediately adjacent to the pericycle, where xylem poles develop, the spatial domain of the model consists of a ring of cells of equal size that can vary only in cell number between simulations (Fig. 4B). In this way, as cell number increases, the size of the ring increases proportionally.

Running the model with uniform initial conditions (i.e. no significant heterogeneity in the input of auxin) results in a spatially homogenous steady state for all three components (Fig. S1). However, in suitable parameter regimes (see supplementary Materials and methods, Figs S2-S6), adding a small amount of noise to the initial level of auxin is sufficient to initiate instability and produce poles of high auxin response. With a configuration of 10 cells, we see one or two xylem poles forming (Fig. 4D), whereas with 20 initial cells, we see either two or three poles (Fig. 4E). This suggests that vascular patterning could be set by small stochastic fluctuations rather than by specific spatial inputs, and supports the concept that such a module can determine pattern as an emergent property.

**Fig. 5.**
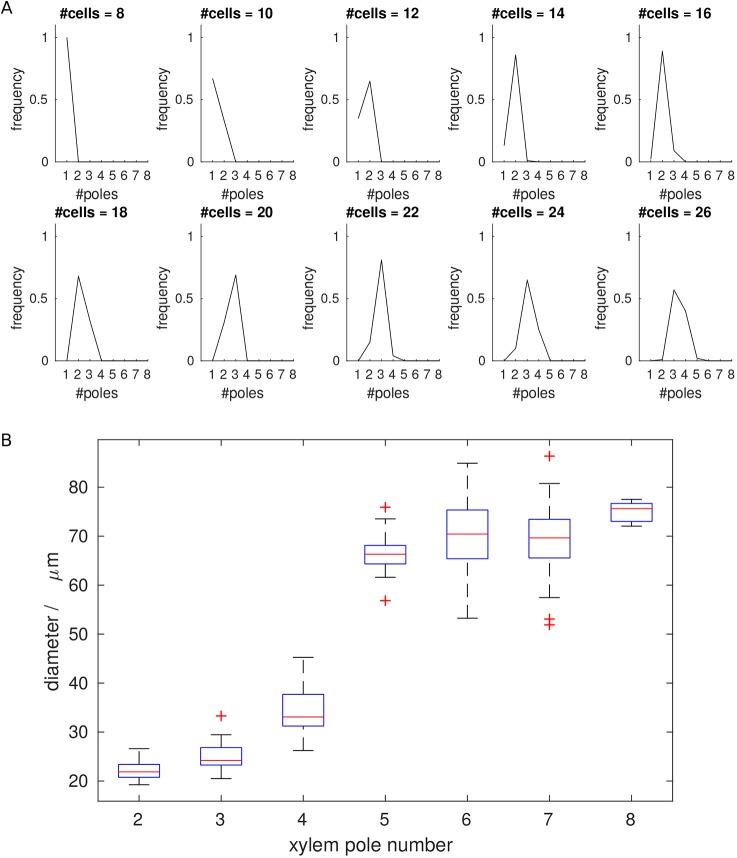
**Vascular patterning is dependent on organ size.** (A) Pole frequency plots for differently sized templates based on 100 simulations. For some sizes, only one pole number is observed, for others there are multiple possibilities. (B) Experimental data showing an increase in xylem pole number in relation to root size for rice. Measurements refer to the diameter of the stele taken between the most marginal xylem-forming cells. In total, 38 crown, 63 lateral and 67 primary roots were analysed. Median values are shown by the red bar and the interquartile range is within the blue box. The whiskers extend to the most extreme data points not considered outliers, while the red + symbols show the outliers (defined as having a distance greater than 1.5 times the inter-quartile range from the edge of the box).

To test the relationship between domain size and pole number further, we ran repeated simulations, using templates with between eight and 26 cells (Fig. 5A). In all simulations, the system appeared to reach a stable steady state, with diverse patterns generated. The smallest template, of eight cells, only ever has one pole. However, for most templates, variation in pole number is observed, i.e. a set of possible outcomes rather than a single solution, with increasing cell number increasing the likelihood of generating more poles. We tested the sensitivity of the model to parameter values by perturbing each of those model parameters that affect steady state in turn (with some parameters such as Hill coefficients combined into groups) (Figs S2-S6). Although changing parameter values altered the exact relationship between cell number and pole count, the overall positive relationship between cell number and pole number is consistent for a broad range of parameter values.

We next investigated whether these observations are representative of the variation seen in plants, using rice, which shows variation in both the size of roots and number of xylem poles, and by sectioning roots and comparing the number of xylem poles with either the diameter of the vascular cylinder or cell number (Fig. 5B and Figs S7,S8). Although the correlation between cell number and vascular diameter is not exact, our data suggest that vascular diameter is reasonably representative of cell number, and from here on we measure only vascular diameter in our experimental analyses. In the original static model, which assumes a constant cell size, this allows direct comparison between data and model. Later on, when we consider division and growth, although cell sizes may vary temporarily, their final sizes are equal so cell number remains directly proportional to diameter.

Consistent with our simulations, we saw an increased likelihood for more vascular poles in the larger roots. As in the model, we observed considerable overlap of possible xylem patterns within roots of particular sizes. For example, roots with a diameter of 74-76 μm could give 5, 6, 7 or 8 poles (Fig. S7). Coupled with the biological observations above, these simulations present a potential mechanism through which distinct patterns could be set in a static (i.e. non-growing) template. In this scenario, the pattern would be determined based on the spatial constraints within which the gene regulatory network operates.

### The heterogeneous input of auxin can override this patterning process to define alternative patterns during embryogenesis

**Fig. 6.**
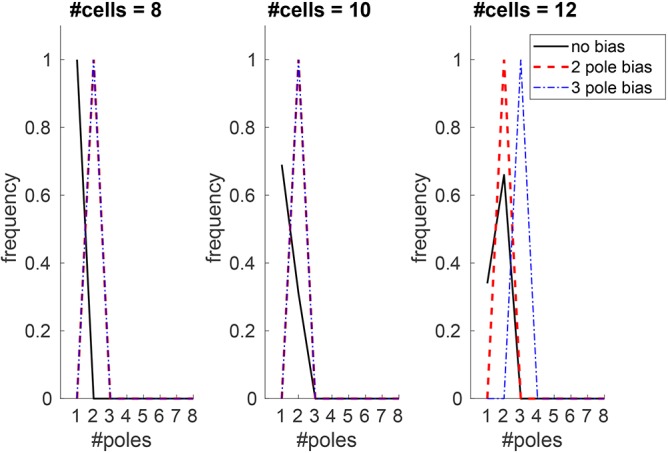
**Heterogeneous input of auxin can establish robust patterns that might otherwise be unstable.** Pole frequency plots for differently sized templates based on 100 simulations. All simulations have stochastic fluctuations in initial auxin according to a normal distribution (see supplementary Materials and methods for details). The black lines represent simulations with uniform auxin production (as in Figs 4 and 5). Red dashed lines represent a persistent bias in auxin production in two opposite cells; blue dot-dashed lines represent a temporary bias in auxin input in three approximately equally spaced cells (owing to the restrictions from using integer values).

The finding that stochastic fluctuations can drive this patterning mechanism to establish stable vascular patterns suggests a module capable of *de novo* pattern formation. However, although this may be the case in some situations, in the *Arabidopsis* embryo, the xylem poles are always positioned relative to the cotyledon primordia (de Rybel et al., 2014), indicating that this initial pattern is not established based on random fluctuations, but depends on the spatial arrangement of the embryo. Although previous models have shown that local inputs of auxin can specify diarch vascular patterns (de Rybel et al., 2014; Mellor et al., 2017), we tested whether this was also the case in our holistic model. We repeated simulations but increased auxin biosynthesis tenfold in two opposite cells within our ring. We found that in all simulations in templates ranging from eight to 26 cells, this resulted in a diarch vascular pattern (Fig. 6 and Fig. S9). This range of sizes included templates where our previous stochastic simulations produced either only one vascular pole or a range of possible outcomes. These results indicate that, when supplied with a localised increase in auxin, a pattern based solely on stochastic elements could be overridden and, in the case of two distinct auxin inputs, specify a diarch vascular pattern.

We therefore propose that this patterning mechanism, which is built around the mutual inhibition of these two hormones, can operate with or without localised inputs to specify different patterns. This would allow flexibility that can drive different patterning outcomes during embryogenesis, influenced by the proximity of the cotyledons in growing plants. We have previously observed that *Arabidopsis* mutants with three cotyledons display an increased number of xylem poles in the hypocotyl (Help et al., 2011). However, it has become apparent that the *phb phv crn athb8* lines used in this study also have an increase in cell number. This raises the issue of whether the extra xylem poles were due to the presence of the third cotyledon or to changes in stele size. To examine this further, we ran simulations as above but with three auxin inputs; these resulted in the establishment of three auxin poles in templates larger than 12 cells (Fig. 6). However, smaller templates (eight and 10 cells) reached a steady-state solution with diarch patterning regardless of whether two or three auxin maxima were included, further supporting the hypothesis that, although an auxin bias can help specify pattern, this influence is also limited by stele size.

### Simulated organ growth provides robustness to vascular pattern

While this static model investigates the relationship between stele size and the number of xylem poles, it does not consider how the pattern evolves in a growing root. Although we see some variation of pole number within some species, for other species this variation does not exist and our static model suggests a greater number of possible outcomes than can be observed experimentally. To address whether organ growth has an effect on stabilizing the patterning process, we incorporated cell growth and division into the model (see supplementary Materials and methods). The growing model simulations have two stages. First, the model is run with a given number of cells (using initial conditions as in the static model described above) with growth and division inactive, to establish an initial steady-state pattern that mimics embryonic development. Second, to simulate post-embryonic growth, the ring is permitted to grow and divide until a specified number and target size of cells is attained. Growth is imposed and limited in this way for convenience, as the aim of the model is not to establish the mechanism by which roots stop and start growing; rather, the aim is to approximate the growth stages and investigate the response of the model given such growth. Division of a given cell is based on the accumulation over time of the product of a generic cytokinin response gene, denoted *R*, modelled in addition to PIN. Once *R* reaches a given threshold, the cell is primed for division. Although PIN and R are modelled in exactly the same way, separating them allows different parameters that determine their sensitivity to auxin and cytokinin to be used. To prevent numerous cells dividing simultaneously, we assign a fixed duration to the mitosis step and allow only one cell to divide at once. This restriction on division was chosen to add a greater degree of irregularity to division, in an effort to better simulate biological variability.

**Fig. 7.**
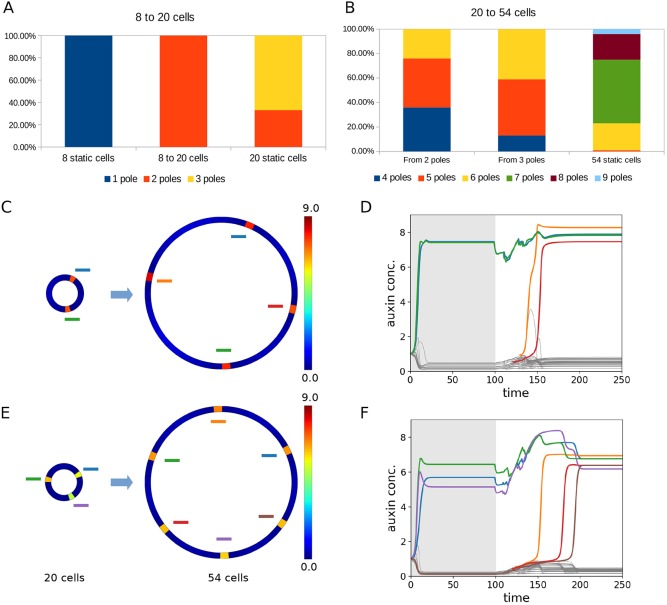
**Growth history plays a key role in determining final organ pattern by stabilising the patterning processes.** (A,B) Comparison of pole frequency in growing versus static 20-cell (A) and 54-cell (B) templates. One-hundred model runs were performed for both growing and static simulations in each case. (C,E) Examples of different final outcomes are shown (right) based upon different initial conditions (left) of templates grown from 20 to 54 cells. The heatmaps in D,F show auxin. In the final solution, each cell is colour coded and the colours are marked on to C and E. The same information can be followed over time (D,F) to reveal the order in which new poles formed.

Using the two-step growth process described above, we ran simulations starting with eight cells (as these produced one pole in 100% of our static simulations) and grew these to 20 cells (as this number of cells produced variable numbers of xylem poles in our static simulations) (Fig. 7A). Whereas static simulations with 20 cells resulted in either two poles (33%) or three poles (67%), when grown from eight to 20 cells, 100% of simulations showed only two poles (Fig. 7A). These predicted differences between the static and dynamic simulations suggest that organ growth can stabilize patterning (i.e. reduce the number of possible outcomes for a given size of template) via a pre-patterning process in the developing organ. We then used the model to test this hypothesis further, by running simulations in which the templates grew larger still. Starting with templates of 20 cells, which have either two or three xylem poles, and growing to 54 cells (this number was selected via trial and error as a number that reliably resulted in an increase in pole number for all pre-growth initial conditions tested), we found differences in behaviour depending on whether the initial 20-cell template had a two- or a three-pole configuration (Fig. 7B-F, Movies 1 and 2). Whereas simulations starting from a two-pole pre-growth configuration resulted in mostly four (36%) or five (40%) poles post-growth, with 24% resulting in six poles, those starting from three poles pre-growth resulted in mostly five (46%) or six (41%) poles post-growth, with only 13% resulting in four poles (Fig. 7B). In general, as may be expected from such a patterning mechanism during growth, although the new poles form approximately midway between existing poles (Fig. 7C,E), there is variation in the time at which new poles appear during and post-growth (Fig. 7D,E). Static simulations for 54-cell templates had between five and nine poles, with seven poles appearing most commonly (Fig. 7B, Fig. S10). In all cases, template growth stabilized the patterning process, reducing the number of xylem poles, although the morphogenetic history/initial conditions also had a significant effect in directing the final patterning outcome (Figs S10 and S11).

### Reduction in organ size reduces xylem pole number

Research in vascular patterning in *Arabidopsis* has focused on the primary root and followed these patterning events from embryogenesis through to the mature root. This is largely due to the availability of marker lines in *Arabidopsis* and to the development of protocols allowing visualisation of these markers during embryogenesis. The lack of such tools in other species makes it exceedingly challenging to follow xylem pole specification during embryogenesis, when the changes in stele size are the greatest. We instead focused on other root types that change size during growth, and imaged crown roots from rice. Crown roots are shoot-borne post-embryonic roots and are established through *de novo* organogenesis. We measured the diameter of the vascular cylinder at the base of the first crown roots of rice at 5 and 8 days post-germination. This data set incorporates ranges from primordia of about 0.5 mm in length to mature roots of over 50 mm in length. When we plotted the length versus the diameter of these roots, we did not see a change in root vascular cylinder diameter (Fig. S12), suggesting that once the primordia forms, the central vascular cylinder does not undergo additional thickening. However, as the root grows, it gradually tapers such that the tip is usually more slender than the base. We performed a more detailed examination of a further 20 roots and made cross-sections through the top, middle and tip of each root. In 19 out of 20 roots, we saw a trend in which the diameter of the vascular cylinder close to the tip was smaller than that at the base, as has been reported in both maize and rice lateral roots (Wu et al., 2016; Sasaki et al., 1981). In our data, 14 out of these 19 roots showed an associated decrease in xylem pole number at the tip compared with the base of the root (Table S1). Similar to the original observation in pea by Torrey, these data show a clear reduction in xylem pole number as the root becomes thinner.

We next asked whether our model would predict similar changes in pole number if the template size and cell number were reduced. To do this, we ran the dynamic model to steady state multiple times with growth and division inactivated, using an initial template of 40 cells, resulting in a set of initial conditions with five to seven xylem poles, approximating the range observed at the base of rice roots. We then reduced the size of this template by reversing the growth process described in the previous section, allowing the roots to gain smaller diameters and, in some simulations, fewer cells. During this process we observed a trend towards fewer xylem poles with decreasing root size, with the largest reductions occurring when both cell number and size are changed (Fig. S13; Movies 3-4). Likewise, starting with templates producing two, three or four poles (20 cells), we could see a shift to either monarch or diarch patterns, similar to those shown previously by Torrey and in our experimental work (Fig. S13).

### Manipulation of stele size determines xylem pole number

**Fig. 8.**
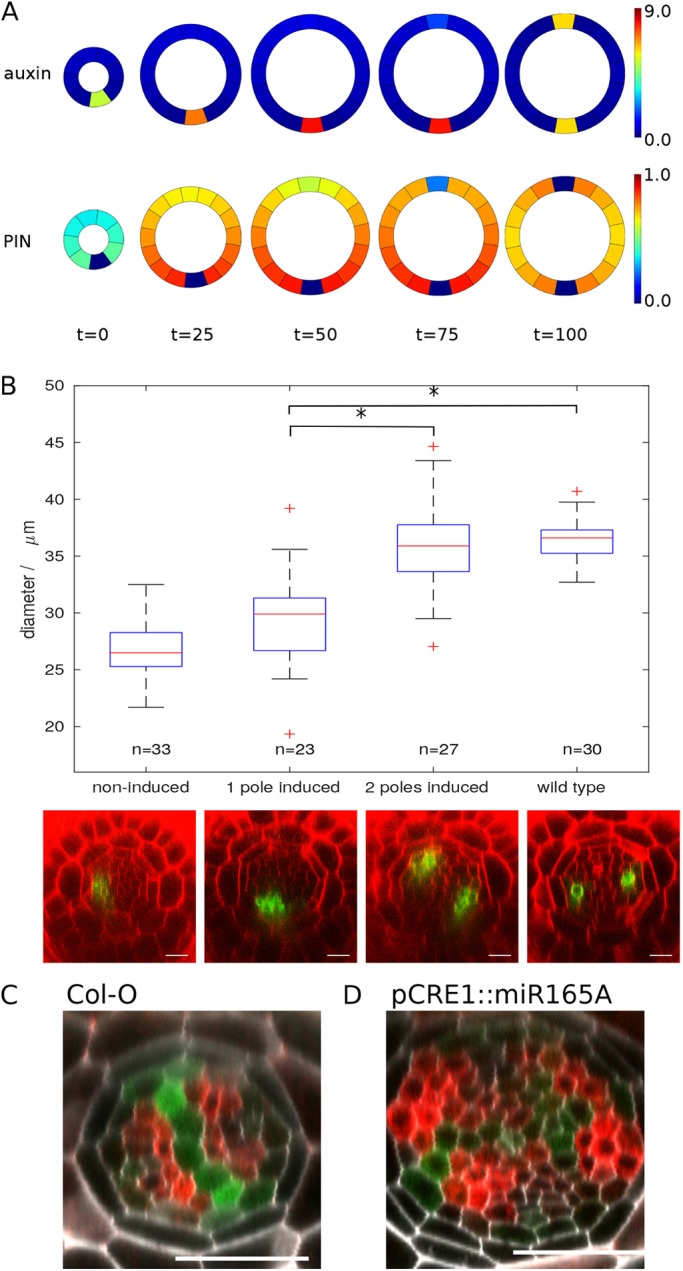
**Stele size has a significant role in modulating the final vascular pattern.** (A) Model simulation of a *lhw* rescue line, showing the level of PIN (bottom) and auxin (top) in the growing template for four time-steps post-simulated LHW induction. (B) Rescue of the *lhw* phenotype in *pCRE1*≫*XVE*≫*LHW* following 72 h induction with β-estradiol*.* The graph shows the relationship between stele diameter (measured here as the internal diameter between pericycle cells) and pole number with representative images shown below. *AHP6::GFP* has been used as a marker for high auxin response and xylem identity, and images have been taken ∼40 μm above the QC. Asterisks indicate results that are statistically different (>95% confidence, Bonferroni corrected *t*-test). (C,D) Increasing stele size results in an increased number of xylem poles. (D) miRNA165A is driven under the *CRE1* promoter, resulting in extra periclinal cell divisions within the stele. The xylem axis can be seen using the IAA2::GFP:GUS marker line and cytokinin response visualised with ARR5::RFP. In this example, four poles of high auxin response can be seen at the perimeter of the vascular tissues. Scale bars: 25 μm.

To provide experimental evidence testing the effect that altering spatial restraints has on vascular patterning, we exploited the *lonesome highway* (*lhw*) mutant in *Arabidopsis* to develop a system in which we can manipulate stele size: *lhw* mutants have a smaller stele than wild-type plants, with just one xylem pole (Ohashi-Ito and Bergmann, 2007). We engineered an oestrogen-inducible rescue line under a stele-specific promoter (*pCRE1*≫*XVE*≫*LHW*). Although in an ideal world we would separate the processes of growth and cytokinin response, they are so intimately related that this cannot be carried out experimentally. However, the above modelling approach allows us to do this readily. To simulate the *lhw* mutant and associated rescue via our transgenic rescue line, we ran simulations with maximum cytokinin production set to 50% of the wild-type level for an allotted period, then reset cytokinin production to 100% for the remainder of the time course to simulate the transgenic rescue of this mutant. We start from eight cells because, as in the *lhw* mutant, simulations run with the static model in this template always produced only one pole with the chosen parameter set. Although the reduction in cytokinin results in weaker expression of PIN, it is still sufficient to drive auxin into a single pole. When the model is permitted to grow under reduced cytokinin conditions, we see no cell divisions; increasing cytokinin production promotes cell division and growth and, if the ring is permitted to grow sufficiently large, a new pole is formed opposite the original one (Fig. 8A, Movie 5). Increasing the ring from eight to 16 or 17 cells resulted in a transition from one to two poles 100% of the time (Fig. S14). Other changes in ring size exhibited the same behaviour, although less robustly.

In our experimental system, uninduced *lhw* plants had an average vascular-cylinder diameter of 26.9 μm, and 32 of 33 plants analysed had just one xylem pole. Following induction with 17-β-estradiol (a synthetic derivative of oestrogen) for 72 h, the diameter of the stele increased and a second xylem pole formed in 54% of plants (Fig. 8B and Fig. S15). We divided the induced plants into two categories based on xylem pole number: those with one pole had an average vascular cylinder diameter of 29.5 µm and those with two poles had an average diameter of 35.9 µm. We performed Bonferroni corrected *t*-test to examine the relationships between vascular cylinder diameter and final pattern: there was no statistical difference between diameter in the two-pole *lhw* rescue lines and the wild-type control, whereas there was a significant difference between one pole-induced and either two pole-induced or wild type. This indicates a clear correlation between pole number and stele size. To further relate xylem pole number to size of the root, we examined the vascular pattern in a transgenic line overexpressing *miR165A* under the *CRE1* promoter (Carlsbecker et al., 2010). These lines showed increased periclinal cell division and consequently had larger vascular cylinders that consisted of more cells. In these lines, we frequently saw additional xylem poles, as determined by the expression of the auxin-responsive IAA2::GFP:GUS marker (Fig. 8C,D). This provides further data supporting the hypothesis that *Arabidopsis* has the potential to re-specify its vascular pattern depending on the spatial constraints of the root.

## DISCUSSION

One-hundred years ago, Claude Wardlaw proposed that stele size was a major factor regulating vascular pole number in roots. For most of the next 100 years, studies into vascular anatomy used a range of different plant species, such as bean, pea and mustard (reviewed in Esau, 1954). However, the molecular studies into the mechanisms controlling vascular patterning over the past 20-30 years have focused almost entirely on the model plant *Arabidopsis thaliana*. The model that has emerged from this species is that, in the primary root, a mutually inhibitory interaction between auxin and cytokinin sets vascular patterning via an input of auxin from the cotyledon apices (Bishopp et al., 2011; De Rybel et al., 2014). Essentially, the two-pole vascular pattern is propagated from the two cotyledons. This diarch vascular pattern is maintained throughout primary growth in *Arabidopsis*.

In this article, we show that the input of hormones from the apical region of the plant is not absolutely required for vascular patterning. This statement is supported both by the *wol* rescue line, in which we show *de novo* establishment of vascular patterning post-embryogenesis, and through simulations where discrete poles of high auxin output can emerge based solely on stochastic perturbations of the initial conditions of the system. However, although we show that input of auxin from the cotyledons is not absolutely required to set the vascular pattern, at least in the case of the *Arabidopsis* primary root, a large body of evidence suggests that this mechanism is used during embryogenesis. By simulating a heterogeneous input, we observe that this can force a diarch vascular pattern in smaller templates that may otherwise give rise to a monarch pattern. We have shown previously that mutants with increased numbers of cotyledons show an increased number of xylem poles in the hypocotyl (Help et al., 2011). Our simulations here confirm this observation and show that, under a wide range of template sizes, three discrete auxin inputs result in three xylem poles. However, the simulations also suggest that, for a sufficiently small template, even with three auxin inputs only two or fewer poles are possible. We propose that the process of pattern propagation from the cotyledon apices to the root pole may play an important role in maintaining robust vascular specification during embryogenesis.

Although vascular pattern is initiated early during embryogenesis, as the stele size increases both during the later stages of embryogenesis and in certain cases post-embryonically, we propose that the initial pattern can be reset into one of the many patterns observed experimentally. Accordingly, simulations run in differently sized templates have a trend towards establishing more poles in larger templates. By manipulating stele size using a transgenic line to rescue the *lhw* mutant, we can see that *Arabidopsis* also has the potential to adapt its vascular pattern to match the spatial constraints of the root. The fact that the diarch vascular pattern is maintained during all stages of primary root growth, most likely reflects the fact that the root never reaches a critical size. The data presented here and elsewhere support a hypothesis that all dicotyledonous plants may establish a diarch vascular pattern initially, and only as the stele size increases would the more characteristic triarch or tetrarch patterns emerge; the general shape of many dicot embryos (such as *Daucus carota* – carrot and *Lactuca sativa* – lettuce) are similar to that of *Arabidopsis* (Esau, 1965). Testing this hypothesis would require the establishment of molecular markers that report early xylem cell identity in a range of dicotyledonous plant species. Although the early events controlling cell fate specification in the root pole of the *Arabidopsis* embryo are well understood and an asymmetry in auxin output has been observed already in the four pro-vascular initial cells, the early events in vascular specification remain unclear in model monocots such as rice. Further work will be needed to identify early xylem markers that can be used to follow cell fate specification in these lines, and determine the effect, if any, that the cotyledon plays in establishing patterning in the primary root in monocotyledonous plants. In other situations, such as during post-embryonic root organogenesis – which makes the vast majority of the root system of monocotyledonous plants – the vascular pattern must be set *de novo*, and this may account for the greater variation in xylem pole number that we observed in rice.

Such a re-patterning process would provide a mechanism whereby final organ size, growth and morphogenetic history can all influence the final pattern. Although we have not looked at the effect of environmental conditions on regulating vascular pattern in this manuscript, it is likely that this forms an important form of regulation in certain contexts. For example, it has been shown that in tropical upland and lowland rice varieties, root traits such as xylem number and diameter are altered when plants are grown in either aerobic or submerged conditions (Kondo et al., 2000). More recently, in *Arabidopsis* it has been shown that either drought treatment or application of the phytohormone ABA caused the formation of extra xylem strands (Ramachandran et al., 2018). This was shown to act via miR165, which regulates levels of both HD-ZIP proteins to tune crosstalk between auxin and cytokinin. A mechanism in which the initial vascular pattern could be re-specified post-embryogenesis would allow such environmental adaptations, either directly, by regulating the gene regulatory networks controlling the specification of xylem as in the case of the application of ABA to *Arabidopsis*, or indirectly, by modulating stele size in rice.

In conclusion, we present a mechanistic model that harmonizes the modern molecular studies with the classic anatomical and physiological studies. By using a simple molecular framework that incorporates key molecules rather than modelling individual components, we are able to apply this to a range of template sizes and observe a trend towards an increase in the number of vascular poles in larger templates. Undoubtedly the exact wiring and individual components that regulate these processes will vary between species, and this will add increased diversity into vascular patterning.

## MATERIALS AND METHODS

### Mathematical model

The static model was implemented in Matlab and the growing model implemented using the Scipy package for the Python programming language. Growth and division are implemented incrementally at each timestep separate from the biochemical model. The counting of auxin poles to produce the frequency plots was carried out using k-means clustering, with the set of auxin values in every cell subdivided into two subsets based on their mean values. The size of the subset with the higher mean auxin was taken to be the number of poles. Auxin promotes cytokinin biosynthesis in the *Arabidopsis* root stele via a linear sequence of transcriptional activation of the transcription factors ARF5/MP, TMO5/LHW and LOG4 (De Rybel et al., 2014). Although we assume that, owing to its small molecular size, cytokinin is free to diffuse from cell to cell, it is known that the movement of auxin depends primarily on the presence of auxin influx and efflux transporters (Bennett et al., 1996; Gälweiler et al., 1998). One such efflux transporter, PIN7 (which we refer to as the more generic ‘PIN’), is known to be upregulated in response to cytokinin (Bishopp et al., 2011). In addition, auxin promotes transcription of AHP6, which interferes with cytokinin signalling. This network is summarised in Fig. 2A. The net result of this is a system in which auxin simultaneously promotes the production of cytokinin while repressing its response. However, as cytokinin can diffuse into neighbouring cells, cells far enough away from an auxin source may be sufficiently free from the repression of the cytokinin response by auxin to produce PIN7. Given that PIN7 acts by actively transporting auxin out of cells, this further aids the establishment of a domain with low auxin and high expression of PIN7 (Fig. 2B). For more details see supplementary Materials and Methods; for parameter values see Table S2.

### Experimental analyses

The *LHW* and CRE1 rescue lines were created using the oestrogen-inducible gateway system described by Siligato et al. (2016). The full coding sequences were cloned from a cDNA template used to generate a second box entry clone. This was combined with the *CRE1::XVE* cassette (Siligato et al., 2016) to give *pCRE1*≫*XVE*≫*LHW* and *pCRE1*≫*XVE*≫*CRE1* and introduced into either the *lhw*-6 mutant carrying the *AHP6::GFP* marker or the *wol* mutant. The *lhw-6* allele was identified in an EMS mutagenesis screen for modifiers of *AHP6* expression (Dettmer et al., 2014) and shown to be allelic to *lhw-1* (Ohashi-Ito and Bergmann, 2007) by transcomplementation. Analyses were performed using a Leica SP5 confocal microscope. For other cross-sections, we used a plastic sectioning approach as described by Mähönen et al. (2000).

## Supplementary Material

Supplementary information
