# Supplementary material for: A core mechanism for specifying root vascular patterning can replicate the anatomical variation seen in diverse plant species
Source: Development. 2019 Mar 15;146(6):dev172411. doi: 10.1242/dev.172411 (PMC6451317; doi:10.1242/dev.172411)
Supplement: Supplementary information [file develop-146-172411-s1.pdf]

Supplementary Materials and Methods

Model Formulation: Dimensional model

To model the production of cytokinin and PIN we use Hill functions. Cytokinin production is an increasing function of auxin, and PIN production is represented by the product of an increasing function of cytokinin and a decreasing function of auxin. We assume a constant production rate of auxin, and linear degradation of auxin, cytokinin and PIN. The movement of cytokinin and auxin between cells is modelled by discretisations of diffusion operators, with auxin transport also dependent on the level of PIN in any given cell.

We denote the concentrations of auxin, cytokinin and PIN in cell i by A_i, C_i and P_i respectively, and use the following dimensional equations:

$$\frac{dA_i}{dt} = \alpha_A - \mu_A A_i + \frac{wD_A}{a_i}\frac{1}{P_{max}}\left(P_{i-1}A_{i-1} - 2P_iA_i + P_{i+1}A_{i+1}\right), \tag{1a}$$

$$\frac{dC_i}{dt} = \alpha_C \frac{\left(\frac{A_i}{\psi}\right)^m}{1+\left(\frac{A_i}{\psi}\right)^m} - \mu_C C_i + \frac{wD_C}{a_i}\left(C_{i-1} - 2C_i + C_{i+1}\right), \tag{1b}$$

$$\frac{dP_i}{dt} = \alpha_P \frac{\left(\frac{C_i}{\phi}\right)^{n_c}}{\left(1+\left(\frac{C_i}{\phi}\right)^{n_c}\right)\left(1+\left(\frac{A_i}{\theta}\right)^{n_a}\right)} - \mu_P P_i, \tag{1c}$$

where $i = 1, 2, ..n$, with n being the number of cells. We set $A_{n+1} \equiv A_1, C_{n+1} \equiv C_1, P_{n+1} \equiv P_0$ so that the model represents a 1-D ring of cells. Production rates are represented by α_A, α_C and α_P with the subscripts A, C and P referring to auxin, cytokinin and PIN respectively. Similarly, degradation rates are represented by μ_A, μ_C and μ_P; ψ, ϕ and θ are binding thresholds, while m, n_c and n_a are Hill coefficients.

The intercellular permeabilities of auxin and cytokinin are represented by D_A and D_C respectively (units ms^{-1}), w is the width of the ring, and a_i is the area of cell i. The constant P_{max} is required for dimensional consistency and we choose for it the maximum possible steady state value of PIN, i.e.:

$$P_{max} \equiv \frac{\alpha_P}{\mu_P}$$

Model Formulation: Nondimensional model

Rescaling concentrations according to

$$A_i \to \frac{\alpha_A}{\mu_A}\overline{A}_i, C_i \to \frac{\alpha_C}{\mu_C}\overline{C}_i, P_i \to \frac{\alpha_P}{\mu_P}\overline{P}_i,$$

and setting

$$D_A \to w\overline{D}_A, D_C \to w\overline{D}_C$$

we have (after dropping the bar notation) the following:

$$\frac{dA_i}{dt} = \mu_A(1 - A_i) + \frac{w^2 D_A}{a_i}\left(P_{i-1}A_{i-1} - 2P_iA_i + P_{i+1}A_{i+1}\right), \tag{2a}$$

$$\frac{dC_i}{dt} = \mu_C\left(\frac{\left(\frac{A_i}{\psi}\right)^m}{1+\left(\frac{A_i}{\psi}\right)^m} - C_i\right) + \frac{w^2 D_C}{a_i}\left(C_{i-1} - 2C_i + C_{i+1}\right), \tag{2b}$$

$$\frac{dP_i}{dt} = \mu_P\left(\frac{\left(\frac{C_i}{\phi}\right)^{n_c}}{\left(1+\left(\frac{C_i}{\phi}\right)^{n_c}\right)\left(1+\left(\frac{A_i}{\theta}\right)^{n_a}\right)} - P_i\right). \tag{2c}$$

In the static case, where all cells have equal size, we define w so that $a_i = w^2$ for all i (i.e. we assume the cross-sectional area of each cell is approximately the square of the contact length between them) and we have:

Development • Supplementary information

$$\frac{dA_i}{dt} = \mu_A(1 - A_i) + D_A\left(P_{i-1}A_{i-1} - 2P_iA_i + P_{i+1}A_{i+1}\right), \tag{3a}$$

$$\frac{dC_i}{dt} = \mu_C\left(\frac{\left(\frac{A_i}{\psi}\right)^m}{1 + \left(\frac{A_i}{\psi}\right)^m} - C_i\right) + D_C\left(C_{i-1} - 2C_i + C_{i+1}\right), \tag{3b}$$

$$\frac{dP_i}{dt} = \mu_P\left(\frac{\left(\frac{C_i}{\phi}\right)^{n_c}}{\left(1 + \left(\frac{C_i}{\phi}\right)^{n_c}\right)\left(1 + \left(\frac{A_i}{\theta}\right)^{n_a}\right)} - P_i\right). \tag{3c}$$

Three key parameters controlling pole spacing are D_A (increasing this enhances the spatial range over which a pole can deplete auxin from its neighbours, thereby enabling larger spacing between poles) and ψ / θ (decreasing both these together effectively increases the overall production rate of auxin, allowing more cells to exceed the theshold concentration, and hence potentially decreasing the pole spacing).

Growth

The increase in the area of cell i (a_i) over time is based on the following ODE:

$$\frac{da_i}{dt} = \eta(a_{max} - a_i)$$

in which η is the growth rate, and a_{max} the maximum cell area. For simplicity, we implement this using the following difference equation:

$$a_i(t + s) = a_i(t) + \eta\ s(a_{max} - a_i(t)), \tag{4}$$

where s is the timestep, and $a_i(t)$ denotes the area of cell i at time t. This leads to a dilution of auxin and cytokinin as the cell grows:

$$A_i(t + s) = \frac{A_i a_i(t)}{a_i(t + s)},$$

$$C_i(t + s) = \frac{C_i a_i(t)}{a_i(t + s)}.$$

To simulate reduction in cell size we simply set a_{max} to be lower than initial cell size in (4) and a negative growth rate follows.

We also modify diffusion and transport of auxin and cytokinin based on current cell sizes in the ODEs as follows:

$$\frac{dA_i}{dt} = \mu_A(1 - A_i) + \frac{D_A}{a_i}\left(P_{i-1}A_{i-1} - 2P_iA_i + P_{i+1}A_{i+1}\right), \tag{5a}$$

$$\frac{dC_i}{dt} = \mu_C\left(\frac{\left(\frac{A_i}{\psi}\right)^m}{1 + \left(\frac{A_i}{\psi}\right)^m} - C_i\right) + \frac{D_C}{a_i}\left(C_{i-1} - 2C_i + C_{i+1}\right). \tag{5b}$$

PIN is membrane bound; we assume that its concentration is unchanged during growth and that the size of the dividing walls remain the same.

Division

The cell division model is threshold based and driven by an additional model component that, like PIN, is cytokinin responsive and inhibited by auxin. We denote this component R_i for the concentration in cell i, and model its evolution over time by the following:

$$\frac{dR_i}{dt} = \mu_D\left(\frac{\left(\frac{C_i}{\xi}\right)^{n_d}}{\left(1 + \left(\frac{C_i}{\xi}\right)^{n_d}\right)\left(1 + \left(\frac{A_i}{\theta}\right)^{n_a}\right)} - R_i\right). \tag{6}$$

Using the same form of equation, but distinguishing it from PIN by introducing the new parameters ξ and n_d, allows for a higher cytokinin threshold for its production to be set. This means that in the *lhw* simulations (see below) the lower level of cytokinin is sufficient to drive weak PIN expression, but not cell divisions.

Each cell has two time-based properties determining whether or not they will divide, denoted 'interphase' (I) and 'mitosis' (M), roughly corresponding to the two biological states after which they are named. Both are initially zero for all cells. We first check if the maximum I is bigger than some threshold (λ_I), and that there are no other cells currently dividing (i.e. that no cells have $M > 0$). If so we increment M a small amount for that cell, and reset I for that cell to zero. Then, for each cell, if M is zero and R is above a given threshold (λ_D)

$$I_i(t+s) = I_i(t) + R_i(t) * s;$$

otherwise:

$$M_i(t+s) = M_i(t) + s,$$

so one and only one of I and M is incremented for a given cell at each timestep.

Finally, if any M exceeds a final threshold λ_M, the cell divides. The area of the two new cells is half that of the parent cell, while the concentrations of auxin, cytokinin, PIN and the cytokin response R remain the same. Mitosis and interphase are reset to zero for both new cells. To mimic the limit on growth that occurs biologically, division is stopped when a certain number of cells is reached.

Reduction in cell size / number

To approximate the evolution of cell lineage over time in tapering roots we run a set of simulations in which both cell size and number are reduced.

To simulate reduction in cell size we simply set a_{max} to be lower than initial cell size in (4) and a negative growth rate follows. In the simulations summarised in Figure S13 we set an initial cell length of 1.5 and a_{max} (in this case a minimum cell length) equal to one.

In addition to a reduction in cell size, we also reduce cell number. This is done by combining a maximum of one pair of cells at each time step until a specified final number of cells is reached. Only cells below a given threshold of $a_i = 1.25$ may be combined, and we choose the cell below this threshold with the lowest level of auxin. This is to ensure that cells which are existing xylem poles do not combine with other cells.

In each case, as with the growing root simulations, we establish an initial steady state with a specified number of cells, before modifying cell size and number.

Timestep schedule and implementation

The static model is implemented using Matlab, with ODEs solved using the ode15s function. The growing model is implemented using the Python programming language, with the ODEs solved using the 'odeint' function in the Scipy package. The ring representations of model results were plotted using the matplotlib package. For the growing model, at each timestep we run

1. the ODE model
2. the growth model
3. the division model

in that order for the specified timestep. Steps two and three can be skipped if the time is less than some 'embryonic time' parameter, allowing the ODE model to get near to steady state before the ring grows.

To produce the pole frequency plots in Figures 3A, S2-S6, and S10-S14 we used a automated loop during which the number of poles were counted using k-means clustering. The set of values for auxin in each cell is split into two subsets based on their mean values using the kmeans algorithm in Matlab (Figures 3A, S2-S6) and Scipy (Figures S10-S14), with the subset with the higher mean value taken contain only the poles. The size of this subset was used as the pole count for that particular run of the simulation.

Simulation of *Lonesome Highway (LHW)* rescue line

To simulate the LHW induction line prior to induction we insert a multiplier (γ) in the production rate of cytokinin in equation (2b) so that:

$$\frac{dC_i}{dt} = \mu_C \left(\frac{\gamma \left(\frac{A_i}{\psi}\right)^m}{1 + \left(\frac{A_i}{\psi}\right)^m} - C_i \right) + \frac{w^2 D_C}{a_i} \left(C_{i-1} - 2C_i + C_{i+1}\right).$$

Setting $\gamma < 1$ in the *lhw* mutant simulation reduces the maximum cytokinin production rate compared to wild type.

To run the model we add an additional time parameter, the 'induction time', to simulate the induction of *LHW* at a given point in time. Experimentally this induction of *LHW* is driven by the introduction of oestrogen. The model runs with γ set to the *lhw* mutant value (< 1) until the 'induction time' is reached. Then, to simulate the induction of *LHW*, γ is increased to one and the production of cytokinin is as in the wild type model.

Parameters and Initial Conditions

The default parameter values used in the models are given in Table S2. Based on observation, largely through trial and error, the Hill coefficients, m, n_c, and n_a must be set greater than two to see any patterning, so we choose the lowest integer value greater than this as the default value. D_A and D_C are the respective permeabilities of auxin and cytokinin which we assume to be roughly equal (when PIN is present in the case of auxin). In order for patterning to occur D_A needs to be sufficiently large when PIN is present in a particular cell to deplete auxin from neighbouring cells. We choose $D_A = 40$ as, when used in conjunction with the other parameters, this results in two poles more often than not for a 14 cell model, roughly equivalent to experimental observation in *Arabidopsis* (Figure S2). The sensitivity parameters ψ, ϕ and θ were also chosen so that patterning would occur with a pole spacing roughly equivalent to that seen in *Arabidopsis*.

Division and growth parameters are selected so that the biochemical model timescales are shorter than those of growth. We assume that the representative division gene is less sensitive to cytokinin than PIN and set $\xi = 0.2$ (compared to $\phi = 0.1$). We assume that peak cytokinin production is reduced by half in the *lhw* mutant so set $\gamma = 0.5$.

Initial conditions of all model components are equal to zero in all cells, except for auxin which has a random distribution of auxin taken from a normal distribution with mean = 1.0 and standard deviation = 0.01. In the growing model the initial size of the cells is also taken from a normal distribution with mean = 1.0 and standard deviation = 0.01. In Figures 7A,B, S10 and S11, where results of static simulations are shown alongside the growing model, we still use the model for auxin and cytokinin given by Equation (5), and retain the initial noise in the cell sizes. However, in these cases we set the growth parameter to zero.

Supplementary Tables

Table S1: **Relationship between stele size and xylem pole number in rice crown roots.** Three sections have been taken from the base of the root, the middle of the root and the root tip. The sections at the base of the root are taken within the first cm, and the sections from the root tip within the first cm. Root diameter is measured between the inner border of endodermal cells. Where there is a reduction in either pole number or diameter between pairs of measurements, these are highlighted with yellow.

Root	Section location	Root Length (cm)	Pole number	Diameter
1	Base	5.8	8	102.956
	Middle		8	82.762
	Root tip		6	68.428
2	Base	6.2	7	85.735
	Middle		6	62.556
	Root tip		6	48.36
3	Base	7.3	6	76.077
	Middle		6	64.678
	Root tip		6	57.199
4	Base	4.7	7	73.849
	Middle		6	66.988
	Root tip		6	65.707
5	Base	9.5	7	84.925
	Middle		6	74.734
	Root tip		6	79.883
6	Base	4.7	7	74.613
	Middle		7	61.848
	Root tip		5	48.305
7	Base	6.8	7	88.169
	Middle		7	71.799
	Root tip		6	63.882
8	Base	5	6	63.216
	Middle		6	76.567
	Root tip		6	65.61
9	Base	5.2	7	82.928
	Middle		6	65.097
	Root tip		6	62.518
10	Base	5.5	6	65.028
	Middle		6	68.195
	Root tip		5	45.076
11	Base	3.5	6	64.823
	Middle		6	64.424
	Root tip		6	58.589
12	Base	5.6	8	107.948
	Middle		8	79.584
	Root tip		6	56.316
13	Base	6.2	6	86.98
	Middle		6	95.232
	Root tip		6	64.073
14	Base	7.1	6	65.111
	Middle		6	58.787
	Root tip		6	53.219
15	Base	6.8	6	65.083
	Middle		6	66.789
	Root tip		5	59.258
16	Base	5.5	6	62.638
	Middle		6	64.007
	Root tip		5	46.863
17	Base	7.1	6	72.199
	Middle		6	58.531
	Root tip		5	60.229
18	Base		6	80.546
	Middle		6	77.317
	Root tip		6	83.061
19	Base		7	65.726
	Middle		6	70.981
	Root tip		6	59.022
20	Base		7	72.488
	Middle		6	75.986
	Root tip		6	68.536

Table S2: Model parameters, with default values.

Biochemical model	
D_A	40
D_C	40
ψ	1.0
ϕ	0.1
θ	1.0
m	3
n_c	3
n_a	3
μ_A	1
μ_C	1
μ_P	1
Growth model	
η	0.1
a_{max}	1.5
w	1
embryonic time	100
Division model	
ξ	0.2
n_d	2
λ_I	3.0
λ_M	1.0
λ_D	0.25
lhw model	
γ	0.5
embryonic time	100
induction time	200

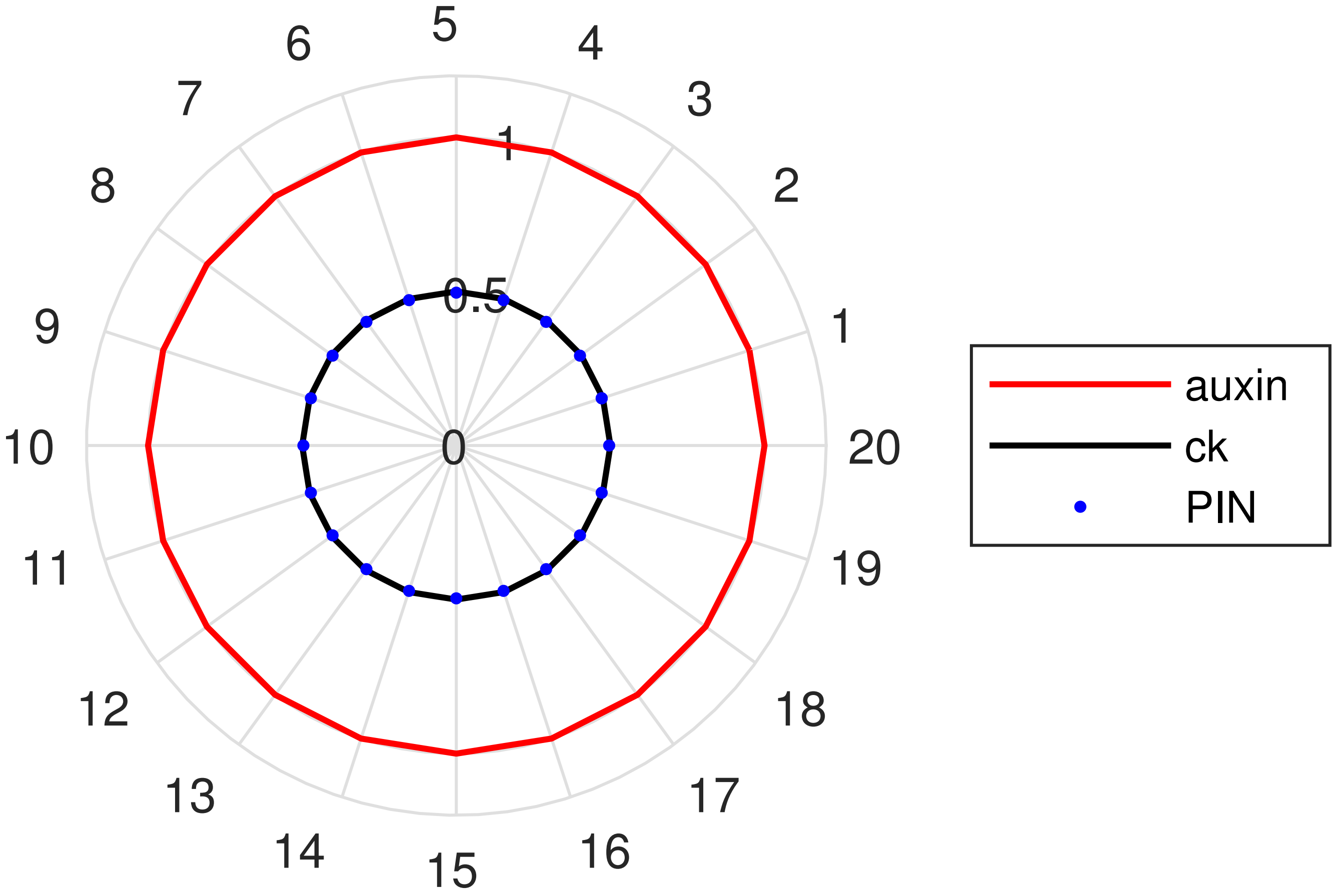

Figure S1: Spatially homogenous steady state of (3a) using parameters in Table S2, with 20 cells and uniform initial conditions (all components equal to one in all cells).

Development • Supplementary information

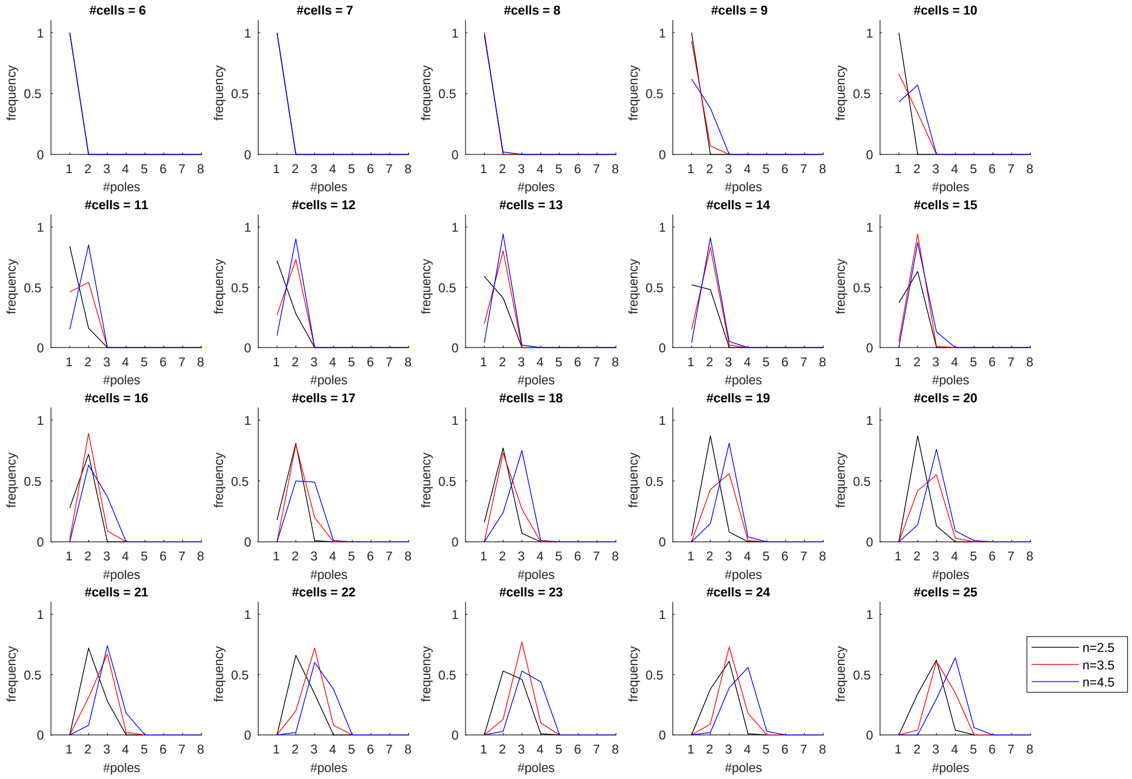

Figure S2: Effect of Hill coefficient n (representing parameters m, n_c, n_a) on pole number frequency distribution with increasing cell number (100 model runs for each cell number with random initial auxin taken from normal distribution with mean=1, s.d.=0.01). All other parameters as in Table S2. In general, increasing n increases the number of poles for a given number of cells. No patterning occurs for $n \leq 2$ (not shown).

Development • Supplementary information

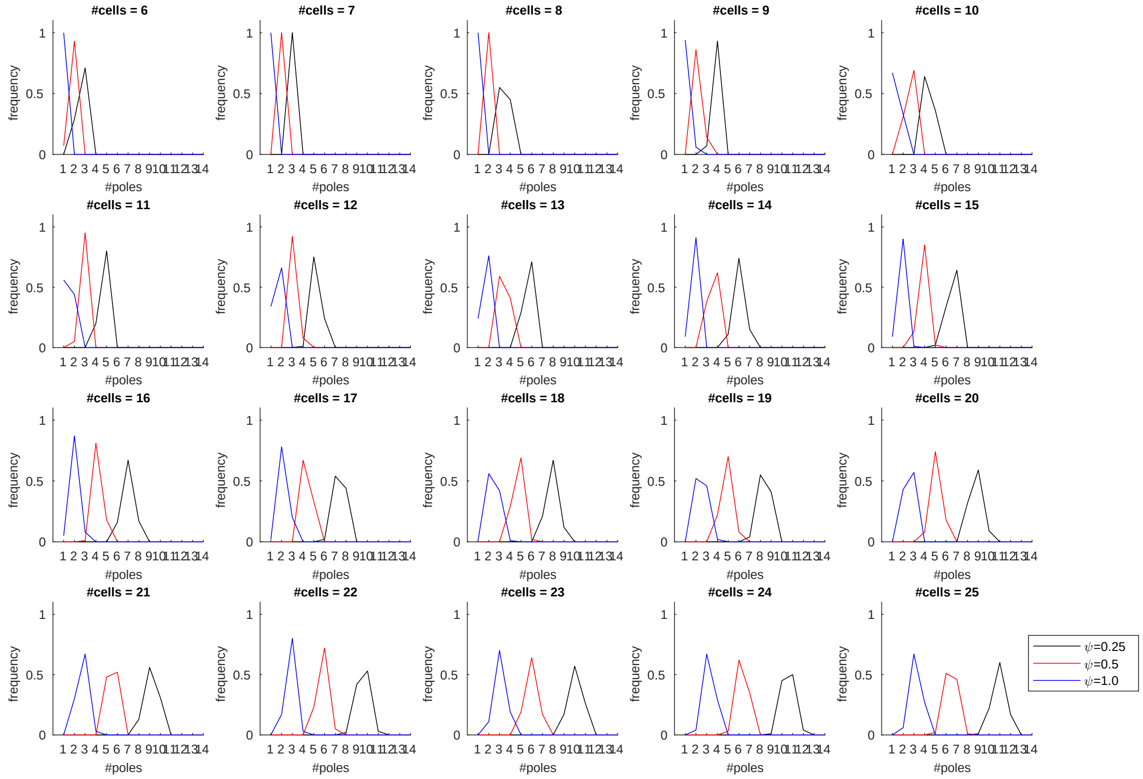

Figure S3: Effect of sensitivity of cytokinin and PIN to auxin (ψ and θ) on pole number frequency distribution with increasing cell number (100 model runs for each cell number with random initial auxin taken from normal distribution with mean=1, s.d.=0.01). All other parameters as in Table S2. In general, decreasing ψ increases the number of poles for a given number of cells. No patterning occurs for $\psi \geq 1.3$ and $\psi \leq 0.1$ (not shown).

Development • Supplementary information

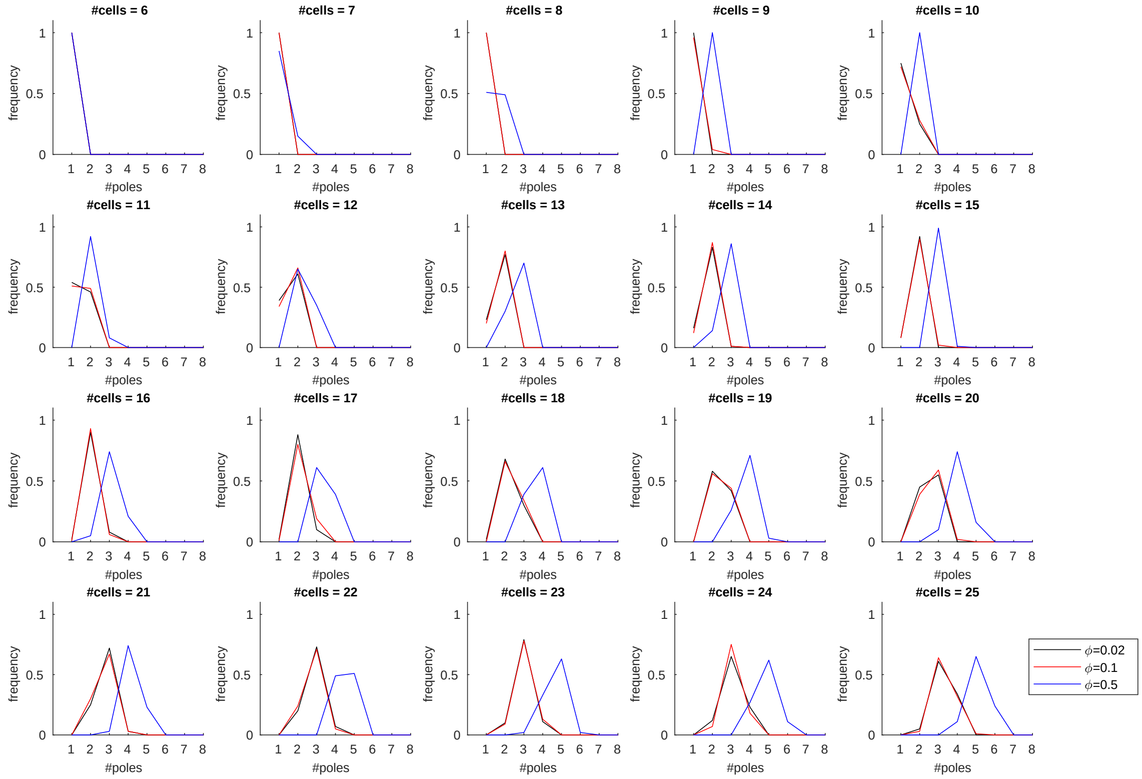

Figure S4: Effect of sensitivity of PIN to cytokinin (ϕ) on pole number frequency distribution with increasing cell number (100 model runs for each cell number with random initial auxin taken from normal distribution with mean=1, s.d.=0.01). All other parameters as in Table S2. In general, decreasing ϕ increases the number of poles for a given number of cells. No patterning occurs for $\phi \geq 1.7$ (not shown).

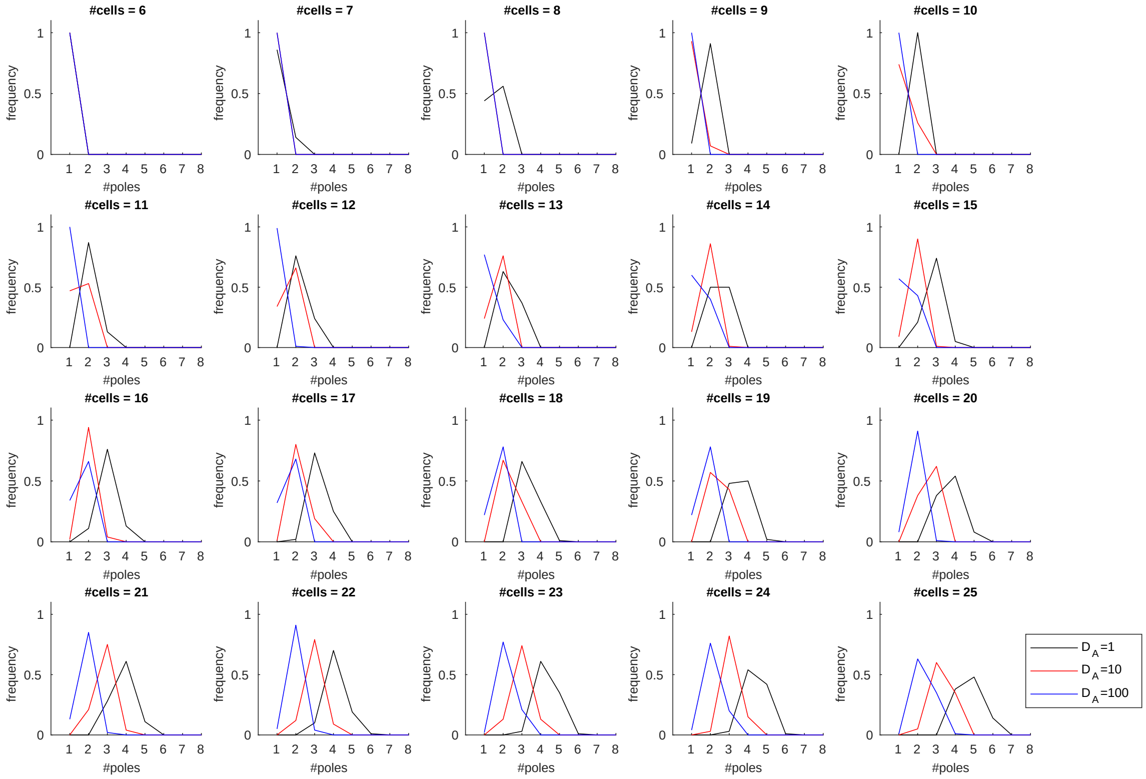

Figure S5: Effect of auxin permeability (D_A) on pole number frequency distribution with increasing cell number (100 model runs for each cell number with random initial auxin taken from normal distribution with mean=1, s.d.=0.01). All other parameters as in Table S2. In general, decreasing D_A increases the number of poles for a given number of cells. No patterning occurs for $D_A \leq 1$ (not shown).

Development • Supplementary information

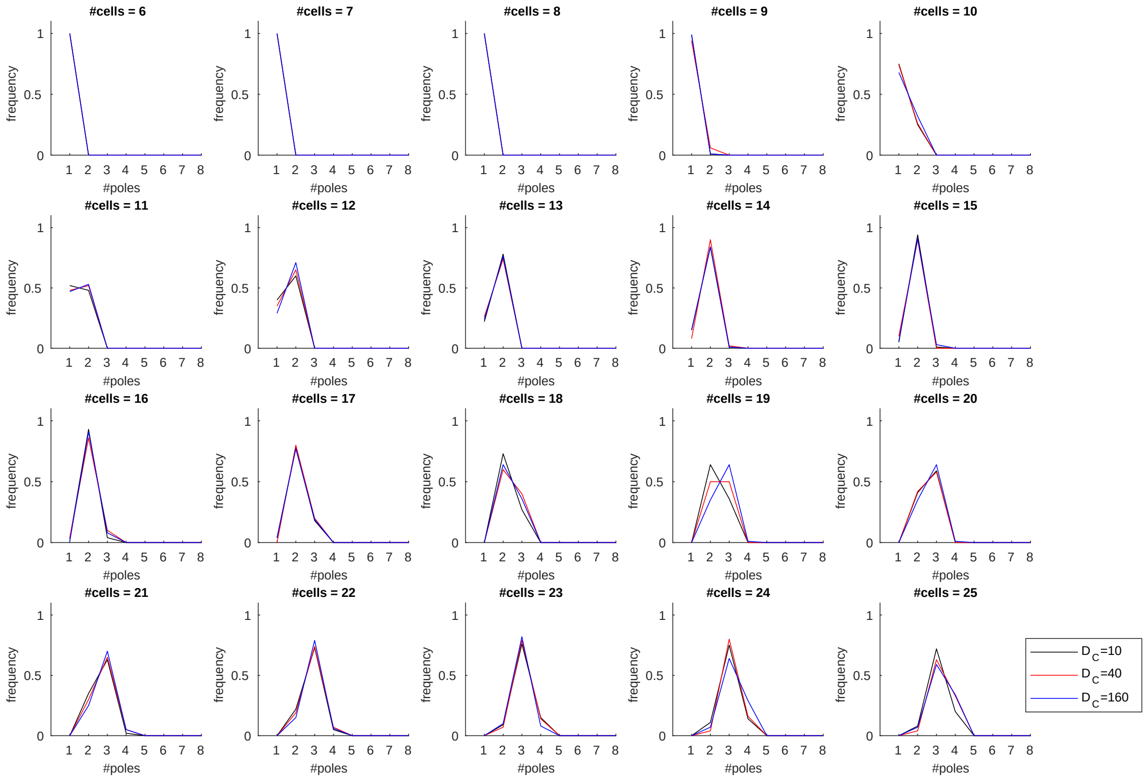

Figure S6: Effect of cytokinin diffusivity (D_C) on pole number frequency distribution with increasing cell number (100 model runs for each cell number with random initial auxin taken from normal distribution with mean=1, s.d.=0.01). All other parameters as in Table S2. In general, changing D_C has no effect on the number of poles for a given number of cells.

Development • Supplementary information

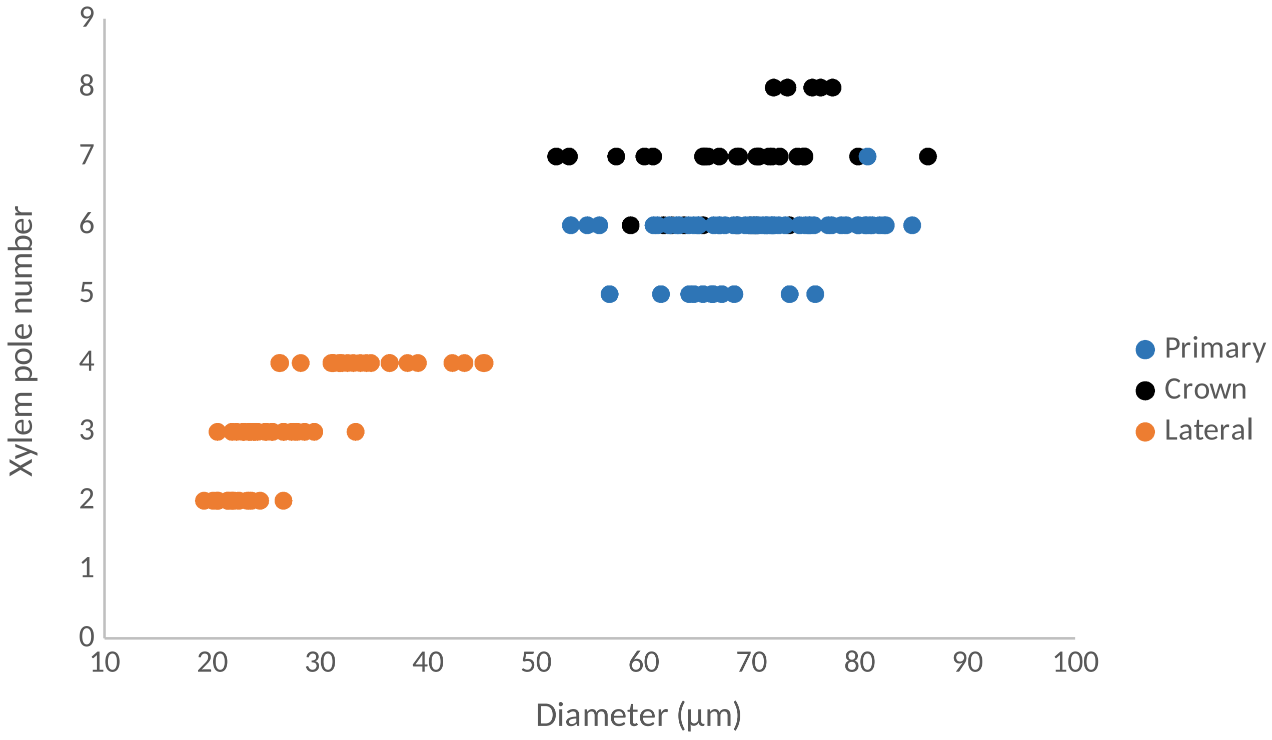

Figure S7: A further breakdown of the data in Figure 5B showing the relationship between root size and xylem pole number for individual rice root types. Diameter refers to the distance between the outer walls of the outermost vascular tissues (i.e. internal to the pericycle).

Development • Supplementary information

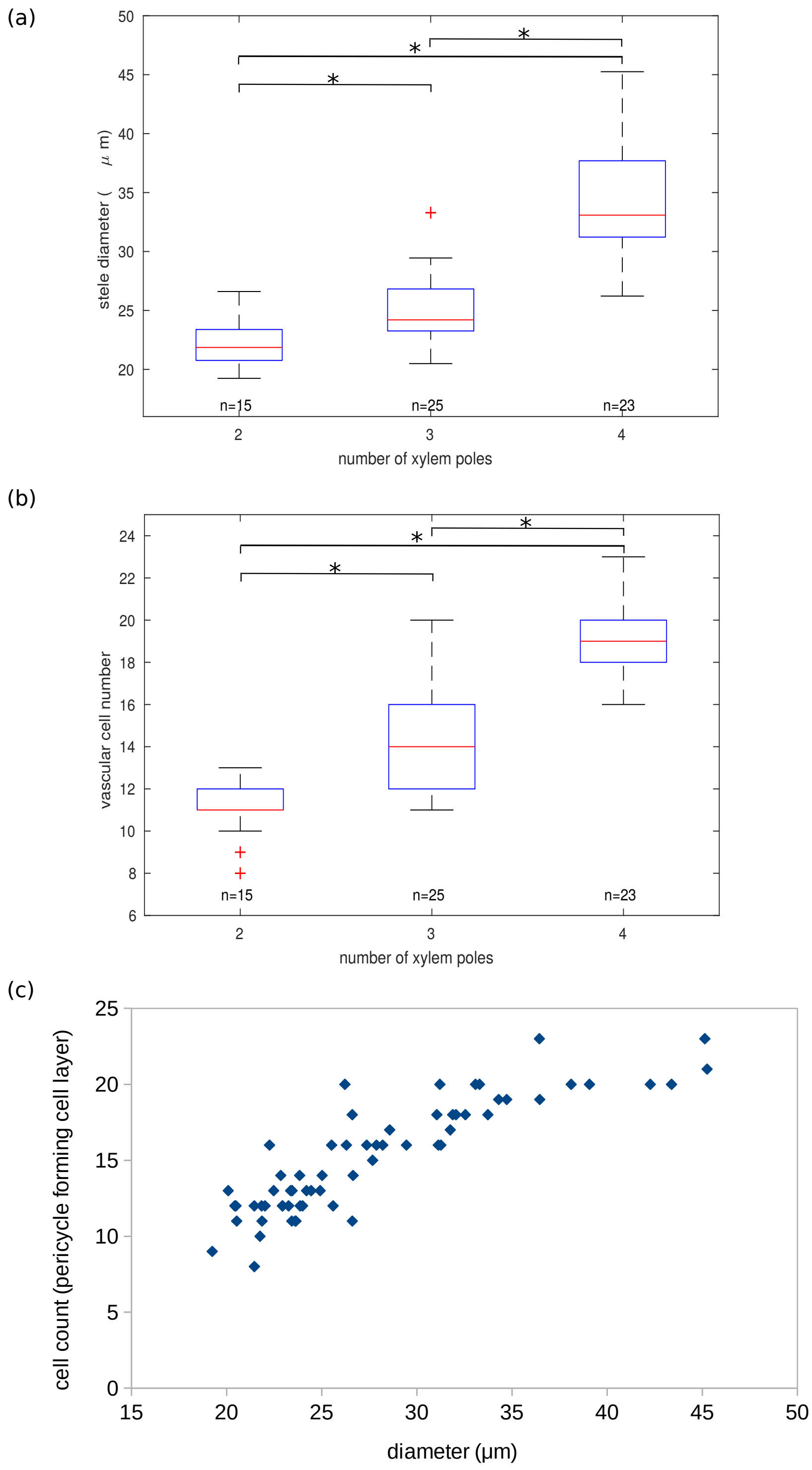

Figure S8: Boxplot showing relationship between (a) stele diameter and xylem pole number, and (b) cell count of vascular cells adjacent to the pericycle layer and xylem pole number, in rice lateral roots. Laterals were sectioned between 0.5 and 1 cm from the root tip. Asterisks and brackets indicate statistically significant pairwise differences (>95% confidence, Bonferroni corrected t-test). (c) Scatter plot showing cell count versus diameter for for data set used to produce (a) and (b).

Development • Supplementary information

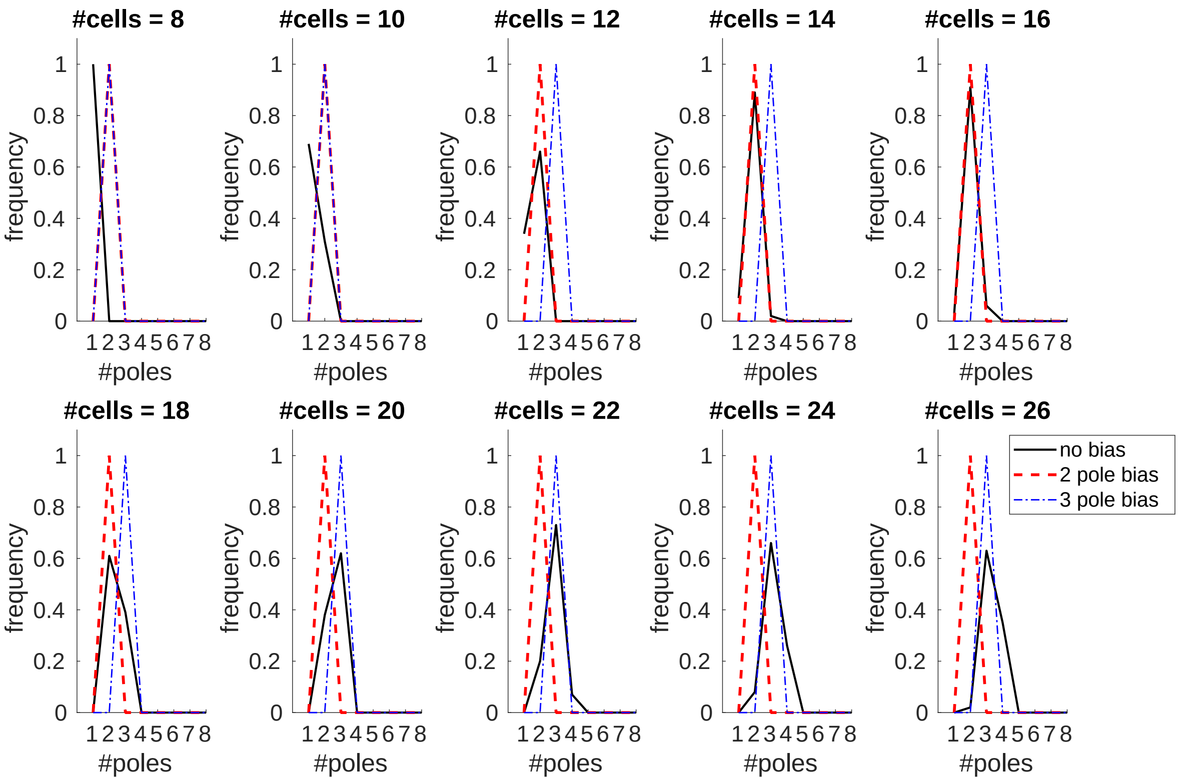

Figure S9: Frequency of pole counts for a range of initial cell numbers using the static model, with no bias in auxin production, a 2-pole bias in auxin production, and a 3-pole bias in auxin production.

Development • Supplementary information

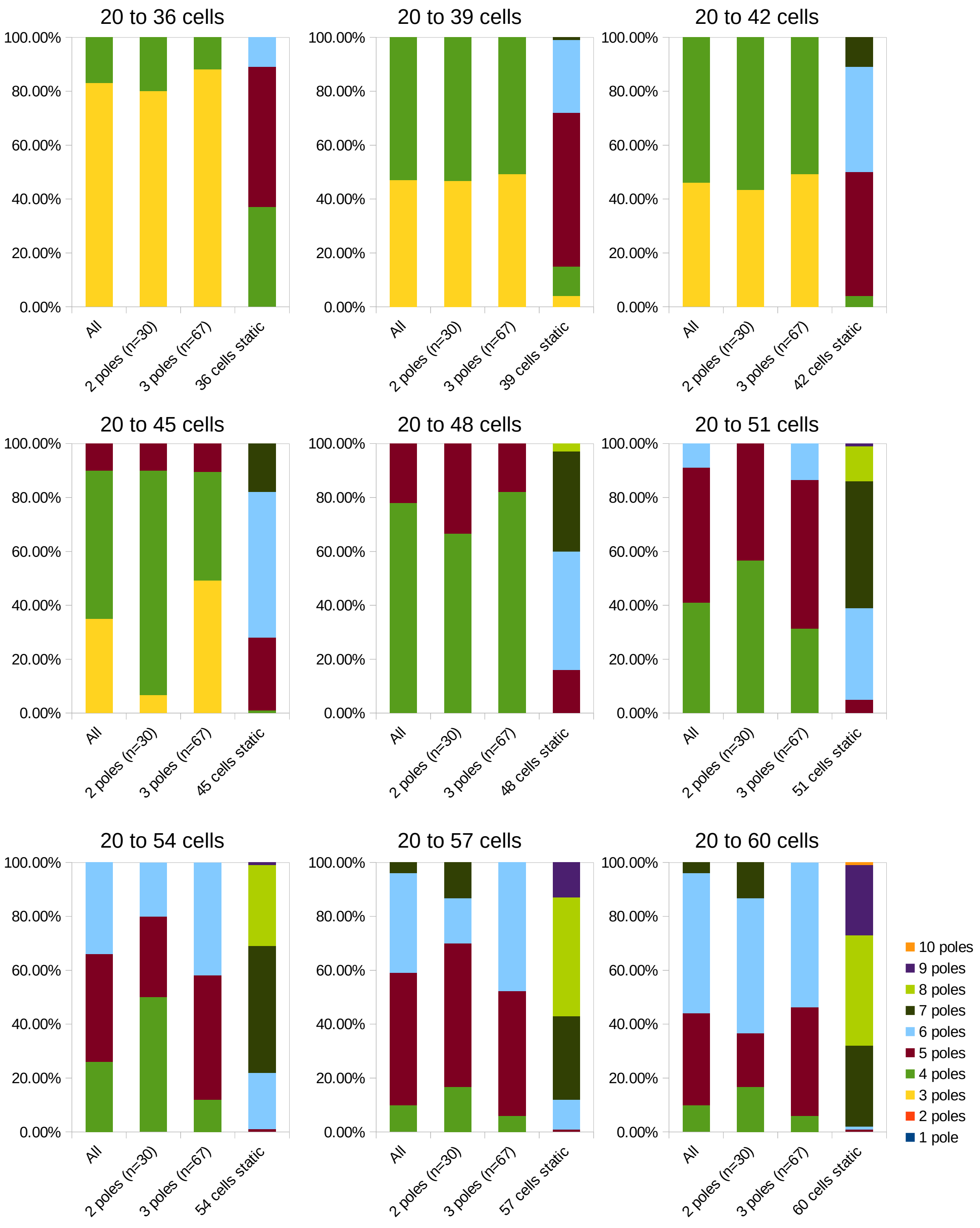

Figure S10: Frequency of poles for the growing ring simulation, starting from a steady state pattern for 20 cells (with no pre-pattern), and growing to a range of final cell numbers (36 to 60 cells). The results are shown for all model runs (n=100), then categorised according to whether the initial 20 cell steady state had two (n=30) or three (n=67) auxin poles (in three cases the initial steady state had 4 poles). Each model was run for 100 time steps to establish steady state, then 200 subsequent time steps with growth and division activated. Also shown for comparison are results for each final cell number, but starting with that cell number with no pre-pattern (n=100). In each case establishing a pattern at a smaller size before growth reduces the ultimate number of poles formed, when compared with the model results in the static template.

Development • Supplementary information

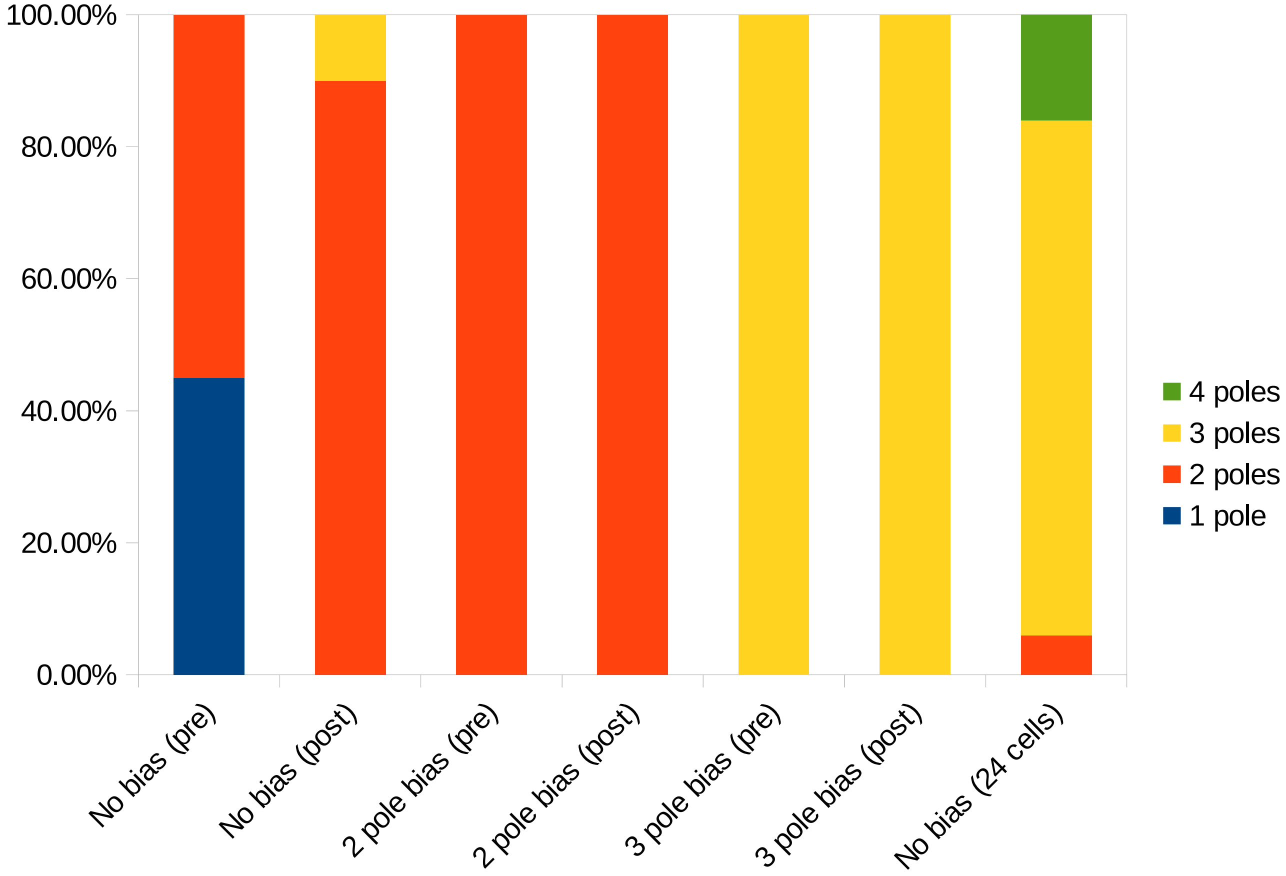

Figure S11: Patterns created through asymmetries in auxin signalling from the cotyledons could be maintained in the growing root. Here, we used an initial template of 12 cells, as this was the smallest template that could produce 2 or 3 poles (based on simulations in Figure S10). We grew this templates to 24 cells and in each case the original pre-pattern was maintained (n=20, each run for 100 time steps to establish steady state, then 200 subsequent time steps with growth and division activated.). In the x-axis labels 'pre' refers to the 12 cell template before growth, while 'post' refers to the state after growth to 24 cells. The final column shows the results for a starting template of 24 cells with no growth or division (n=100).

Development • Supplementary information

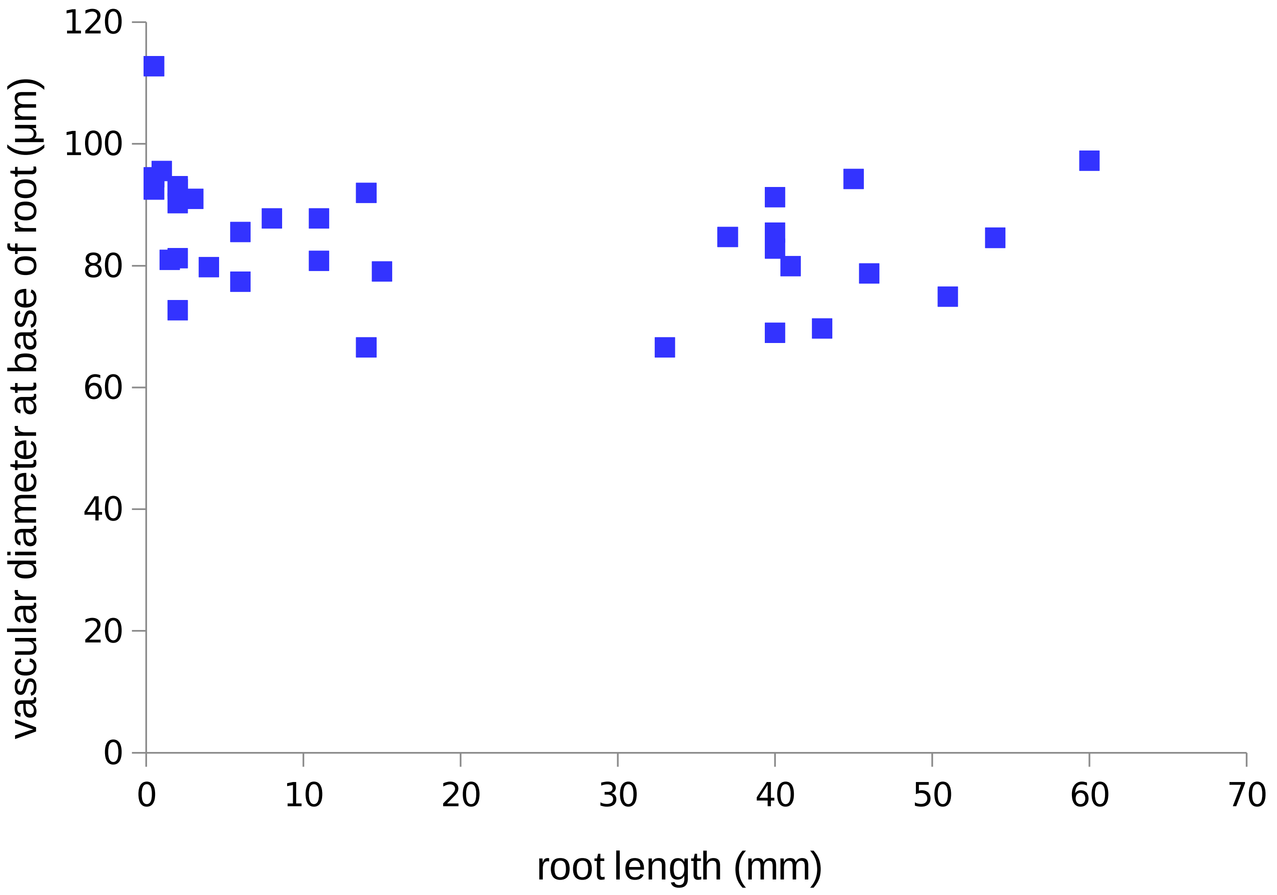

Figure S12: Vascular diameter at base of root versus root length of first embryonic crown roots from rice plants 5 days and 8 days after germination. This data supports a model where the diameter of the vascular tissue remains unchanged at the base as the root grows from a 5mm primordium to a root of up to 70mm. Within roots of this length we see a clear tapering to the root tip (see also Table S2). We grouped primordia of less than 2mm together and saw an average of 6.4 ± 0.5 xylem poles. This is similar to the rest of the data set, where we see an average of 6.7 ± 0.7 xylem poles.

Development • Supplementary information

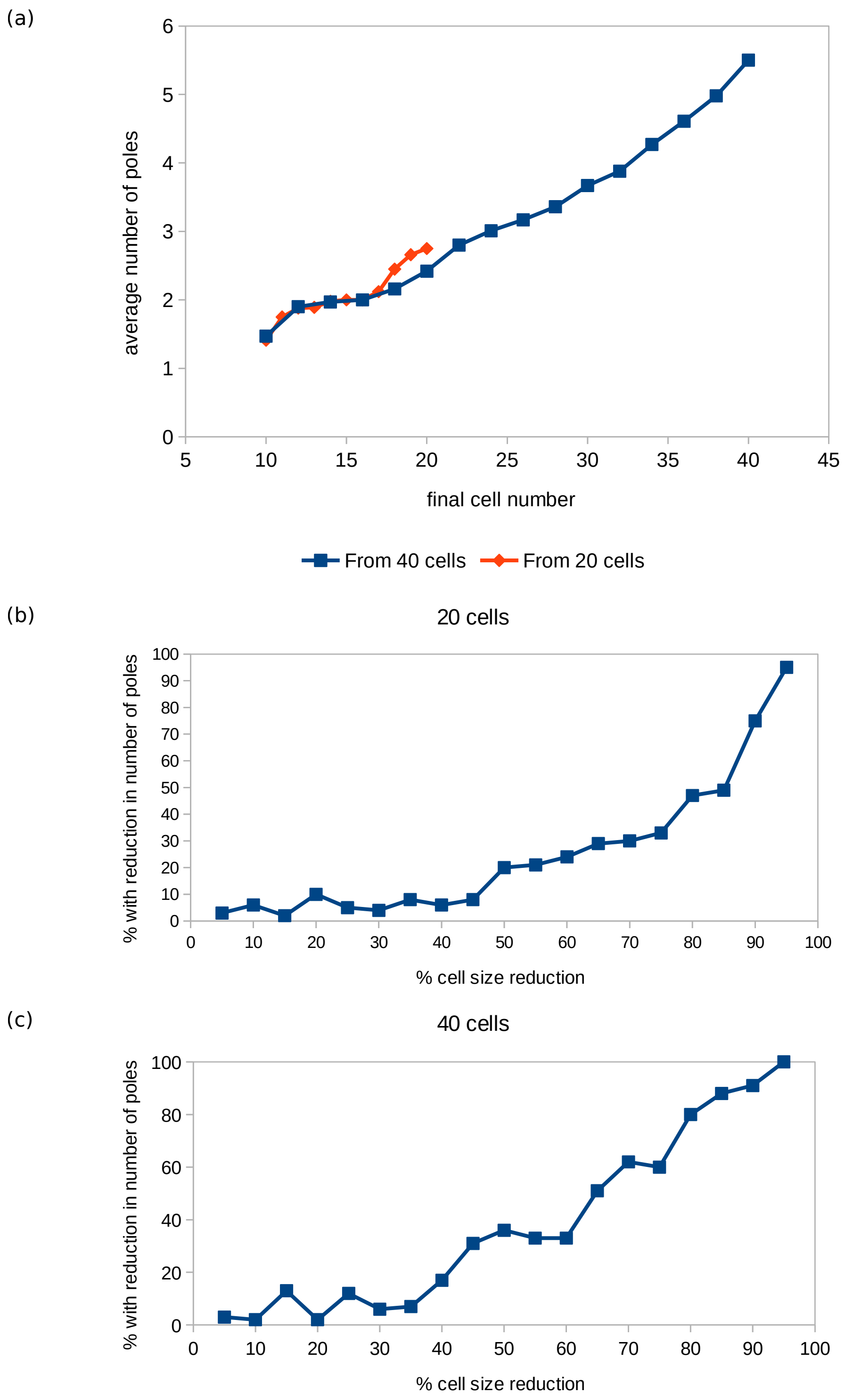

Figure S13: (a) Average number of poles versus final cell number in simulations in which both the cell number and size are reduced from initial templates of 40 and 20 cells (approximating the evolution of cell lineage over time in tapering roots). See section for description of vascular size reduction. 100 repeats, each run for 100 time steps to establish steady state, then 200 subsequent time steps with growth and division reversed. (b,c) Reduction in cell size only, no reduction in cell number. Percentage of roots showing a reduction in pole count versus percentage reduction in size from initial size of 2. (a) Initial cells = 40. (b) Initial cells = 20.

Development • Supplementary information

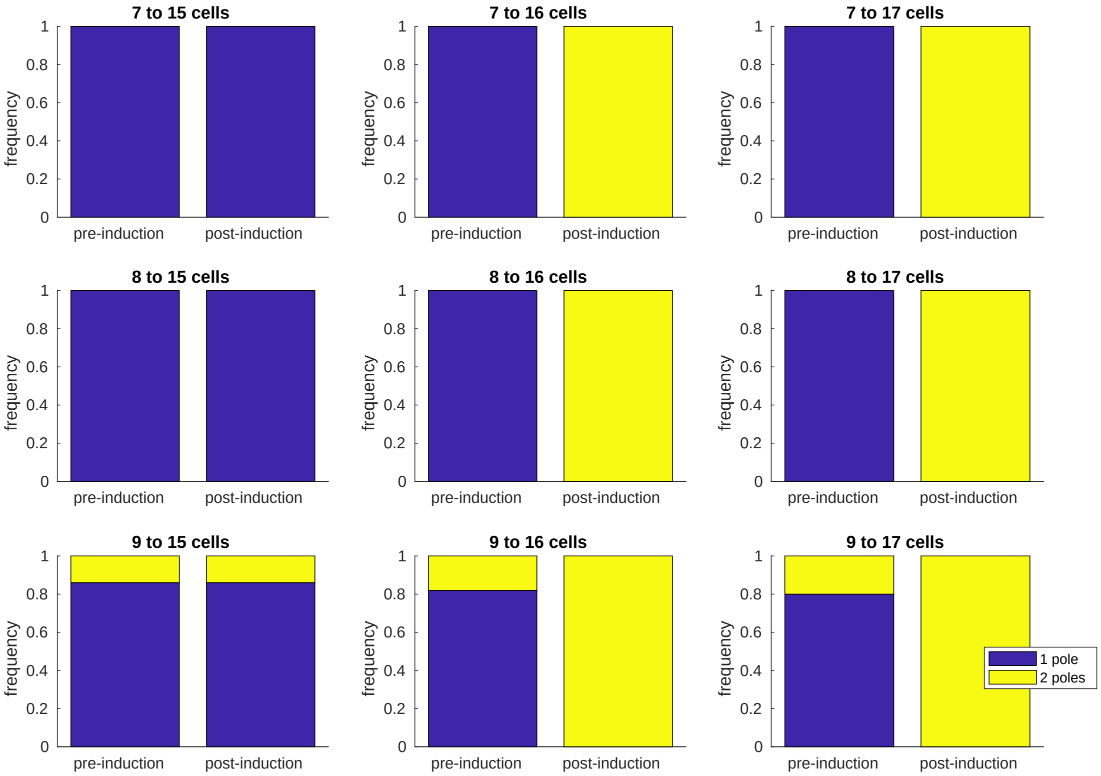

Figure S14: Frequency of poles for the *lhw* rescue simulation (see section), pre- and post- simulation LHW induction for a range of cell number transitions during growth. 100 model runs for each subplot, each run for 100 time steps pre-growth, then a further 100 time steps with growth but no LHW increase, then a final 100 time steps with both a LHW increase and growth possible. Increasing the ring size to 15 cells is insufficient to generate another pole, while starting from 9 cells results in two poles pre-induction around 10-20% of the time. Otherwise the model changes from one to two poles every time.

Development • Supplementary information

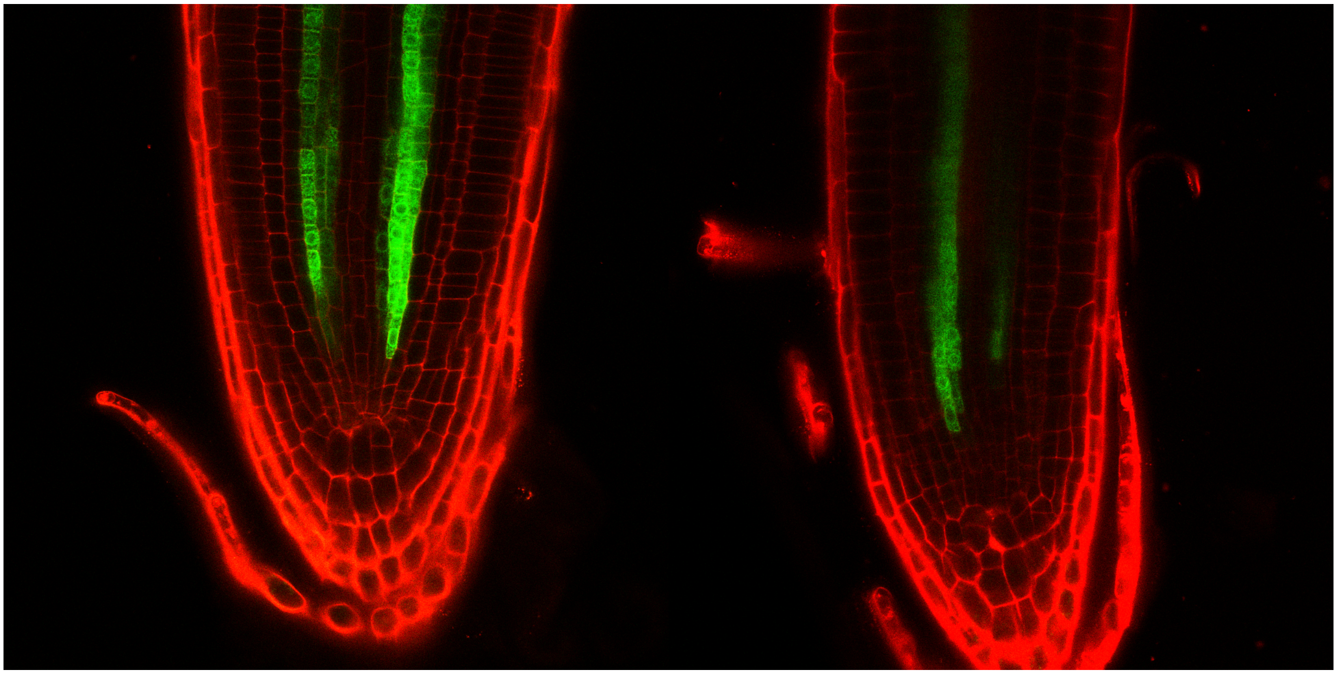

Figure S15: Longitudinal images of *lhw* rescue line. Here AHP6::GFP is clearly present in two distinct poles that do not connect. In the image on the right, only a few cells express AHP::GFP in one of the poles.

Development • Supplementary information

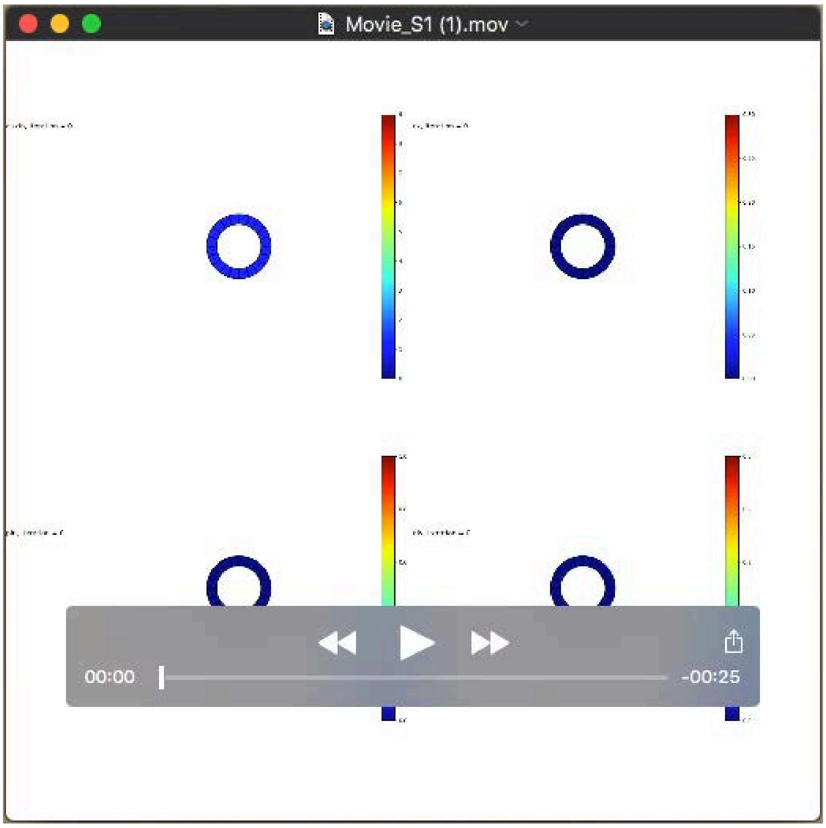

Movie 1
Animation showing the time course (250 time steps) of auxin, cytokinin (ck), PIN (pin) and the generic cytokinin response gene that drives division (div) in a model run starting from 20 cells in which a steady state with *two* auxin poles is formed before growth begins. After growth and division up to 54 cells is activated (at 100 time steps) the model evolves to a new steady state with *four* poles. See also Figure 7C,D for plots from the same simulation.

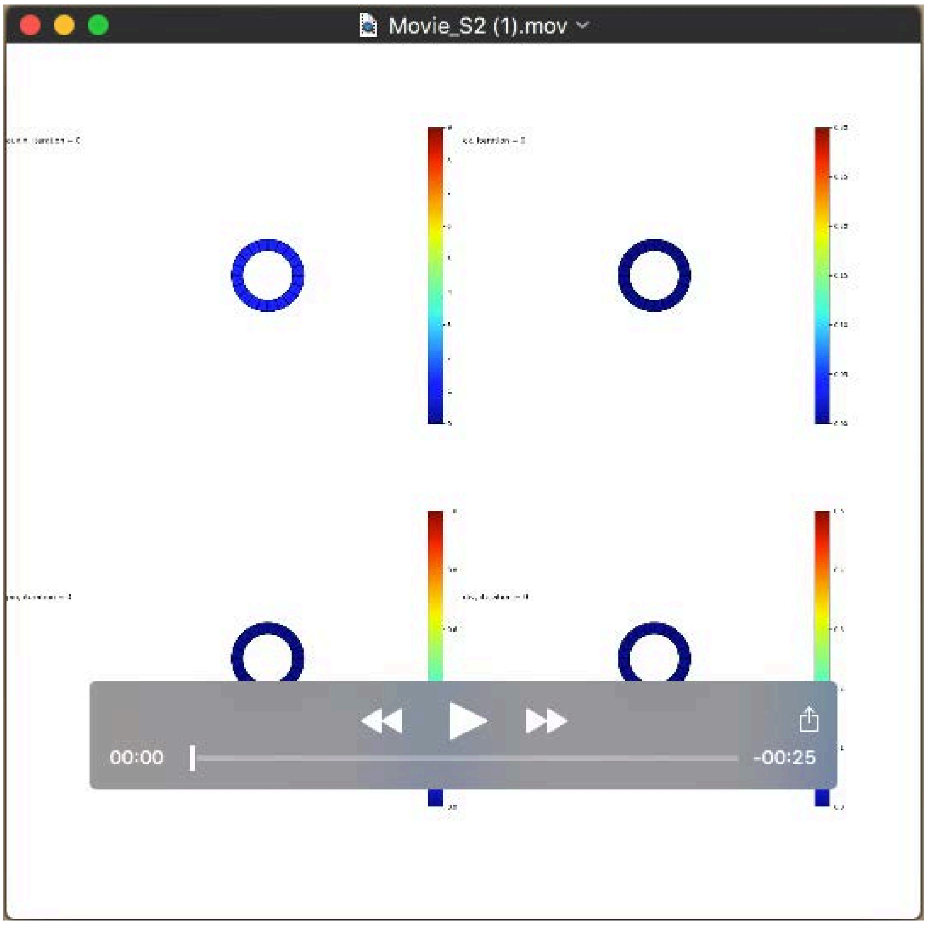

Movie 2
Animation showing the time course (250 time steps) of auxin, cytokinin (ck), PIN (pin) and the generic cytokinin response gene that drives division (div) in a model run starting from 20 cells in which a steady state with *three* auxin poles is formed before growth begins. After growth and division up to 54 cells is activated (at 100 time steps) the model evolves to a new steady state with *six* poles. See also Figure 7E,F for plots from the same simulation.

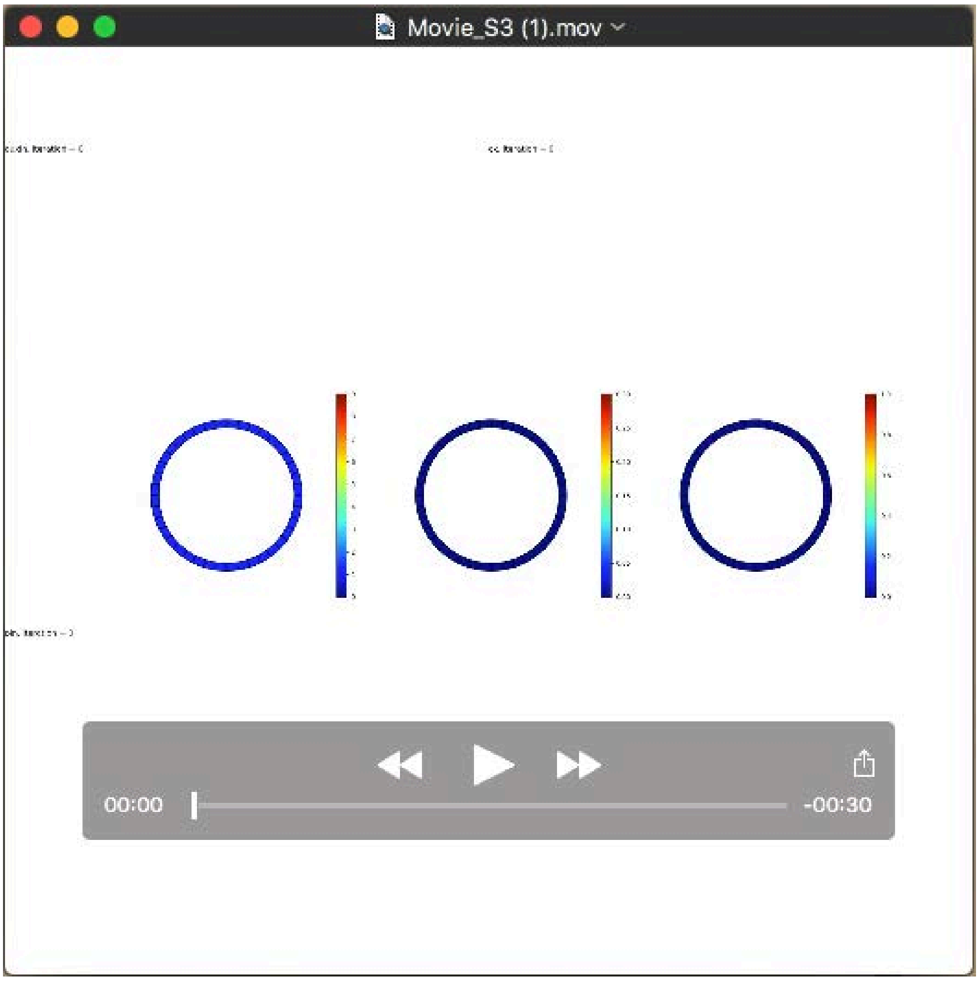

Movie 3
Animation of model time course (300 time steps) showing auxin (left), cytokinin (centre) and PIN (right), in which cell number and size are reduced from 40 to 30 cells (at 100 time steps), after being allowed to reach a steady state in the larger 40 cell template. After the reduction in size and cell number the number of poles also reduces from seven to four.

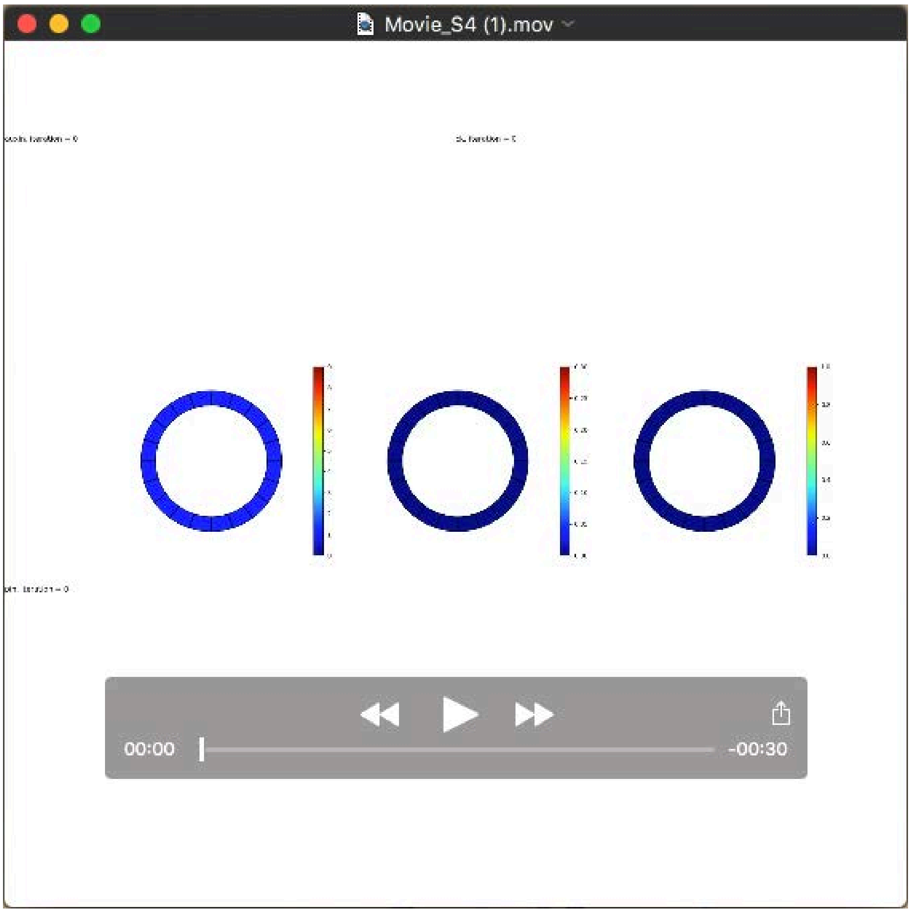

Movie 4
Animation of model time course (300 time steps) showing auxin (left), cytokinin (centre) and PIN (right), in which cell number and size are reduced from 20 to 10 cells (at 100 time steps), after being allowed to reach a steady state in the larger 20 cell template. After the reduction in size and cell number the number of poles also reduces from two to one.

Development • Supplementary information

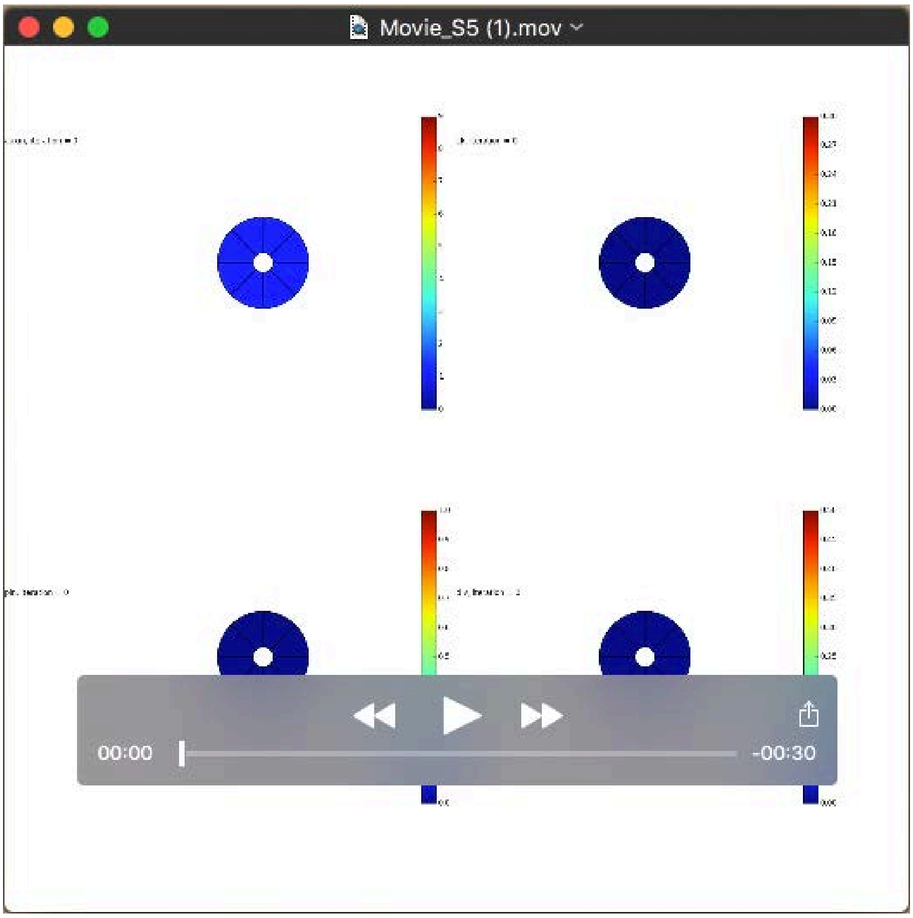

Movie 5

Animation time course (300 time steps) showing auxin, cytokinin (ck), PIN (pin) and the generic cytokinin response gene that drives division (div) in the simulation of the *LHW* induction line. Starting from an eight cell template, growth is activated after 100 time steps, then to simulate the induction of *LHW* the production rate of cytokinin is increased after 200 time steps for the remainder of the time course. The induction of *LHW* promotes cell division, growth, and the formation of an additional xylem pole. Growth is limited by limiting the number of cells at 16.
